# Global prevalence of asymptomatic norovirus infection in outbreaks: a systematic review and meta-analysis

**DOI:** 10.1186/s12879-023-08519-y

**Published:** 2023-09-12

**Authors:** Jun Wang, Zhao Gao, Zu-rong Yang, Kun Liu, Hui Zhang

**Affiliations:** 1 Department of Clinical Laboratory, Jiaozhou Central Hospital, 29 Xuzhou Road, Qingdao, Shandong 266300 P.R. China; 2 Department of Clinical Laboratory, Jinan Second Peoples’ Hospital, 148 Jingyi Road, Jinan, Shandong 250000 P.R. China; 3 Department of Epidemiology, Ministry of Education Key Lab of Hazard Assessment and Control in Special Operational Environment, School of Public Health, Air Force Medical University, 169 Changle west Road, Xi’an, Shaanxi 710032 P.R. China; 4 Department of Prevention of Infectious Diseases, Xi’an Center for Disease Control and Prevention, 599 Xiying Road, Xi’an, Shaanxi 710054 P.R. China

**Keywords:** Norovirus, Outbreaks, Asymptomatic, Prevalence, Meta-analysis

## Abstract

**Background:**

Although many studies on asymptomatic norovirus infection in outbreaks have been conducted globally, structured data (important for emergency management of outbreaks) on the prevalence of this epidemic are still not available. This study assessed the global prevalence of asymptomatic norovirus infection in outbreaks.

**Methods:**

We identified publications on asymptomatic infections from norovirus outbreaks by searching the PubMed, Embase, Cochrane Library, Medline, and Web of Science databases and screening references from the articles reviewed. Prevalence of asymptomatic norovirus infection in outbreaks was employed as the primary summary data. The random-effects model of the meta-analysis was fitted to generate estimates of the prevalence in the overall and subgroup populations.

**Results:**

In total, 44 articles with a sample size of 8,115 asymptomatic individuals were included. The estimated pooled prevalence of asymptomatic norovirus infection in outbreaks was 21.8% (95%CI, 17.4–27.3). The asymptomatic prevalence of norovirus GII (20.1%) was similar to that of GI (19.8%); however, the proportion prevalence of asymptomatic individuals involved in the former (33.36%) was significantly higher than that of in the latter (0.92%) and the former (93.18%) was reported much more frequently than the latter (15.91%) in the included articles. These studies had significant heterogeneity (I^2^ = 92%, τ^2^ = 0.4021, P < 0.01). However, the source of heterogeneity could not be identified even after subgroup analysis of 10 possible influencing factors (geographical area, outbreak settings, outbreak seasons, sample types, norovirus genotypes, transmission routes, subjects’ occupations, subjects’ age, per capita national income, and clear case definition). Meta-regression analysis of these 10 factors demonstrated that the geographical area could be partly responsible for this heterogeneity (P = 0.012).

**Conclusions:**

The overall pooled asymptomatic prevalence of norovirus in outbreaks was high, with genome II dominating. Asymptomatic individuals may play an important role in norovirus outbreaks. This knowledge could help in developing control strategies and public health policies for norovirus outbreaks.

**Supplementary Information:**

The online version contains supplementary material available at 10.1186/s12879-023-08519-y.

## Background

Norovirus (NoV) is the leading causative agent of both sporadic and acute gastroenteritis (AGE) outbreaks in all age groups. It accounts for 16–18% of all AGE cases globally and causes an estimated 685 million illnesses, resulting into 210,000 deaths and 15 million disability-adjusted life years annually [1–3]. In recent years, many countries have been experiencing an increase in AGE outbreaks associated with NoV, the second largest burden of all infectious diseases worldwide [2, 4, 5]. At present, norovirus GI and GII variants are the most frequently detected genomes worldwide. In particular, GII.4, a genotype of GII, is the most prevalent variant [6]. The following factors are responsible for the highly contagious nature of NoV: low infectious dose [7], short duration of immune protection, viral shedding for a long duration (≥ 1–3 weeks [8–12]) in both symptomatic and asymptomatic individuals, stability of the environment, and multiple routes through which the pathogen can reach the human host (i.e., person-to-person contact, food, water, and aerosols) [13]. Within 12–48 h of exposure to these small, non-enveloped, single-stranded RNA viruses that cause this self-limiting disease, most people generally experience nausea, vomiting, watery diarrhea, stomach cramps, headache, and fever, which last for approximately 3 days [13]. At present, there are no antiviral or specific therapeutic drugs for NoV, and vaccines are still under development and have not been approved for marketing [14]. In addition, limited measures are available to prevent and control NoV outbreaks and they mainly include health intervention, isolation of infected people (including asymptomatic individuals), disinfection of equipment, infrastructure, objects, etc. with chlorine-containing disinfectants, and enhanced hand and personal hygiene [10]. However, the level of isolation needs to be carefully considered to avoid affecting the normal lives of other healthy people. Therefore, it is necessary to gain more insight into the asymptomatic infection caused by NoV in outbreaks.

Many reports have shown that AGE outbreaks can be caused by the excreta of asymptomatic individuals infected with NoV [15–17]. The duration and amount of fecal virus shedding from asymptomatic NoV carriers were similar to or slightly lower than those from symptomatic patients [8, 18]. As a result, it is extremely difficult to control NoV outbreaks [17], especially with asymptomatic food handlers [15, 16]. Prevalence of asymptomatic NoV infection varies across outbreaks. A study on NoV in outbreak settings in Spain reported an asymptomatic prevalence rate of 54% among food and healthcare workers exposed to the virus [11]. A long-term surveillance of NoV outbreaks in food and dining establishments in Japan reported an asymptomatic prevalence of approximately 7% [19]. The asymptomatic prevalence of NoV outbreaks in long-term care facilities in the United States was 12% [20]. A report from Shanghai, China, showed an asymptomatic prevalence of 14.4% for NoV in outbreaks [21]. However, no meta-analysis has yet been conducted to specifically address the prevalence of asymptomatic NoV in outbreaks. Understanding the prevalence of asymptomatic NoV infection in outbreaks can facilitate the formulation and application of successful public health control policies. This study aims to summarize the overall prevalence of asymptomatic NoV infection in outbreaks. It then assesses the prevalence through different subgroup variables (geographic area, outbreak settings, outbreak seasons, sample types, NoV genotypes, transmission routes, subjects’ occupations, subjects’ age, per capita national income, and clear definition of asymptomatic individuals and symptomatic cases).

## Methods

This systematic review and meta-analysis was conducted and reported in accordance with the Meta-analyses Of Observational Studies in Epidemiology (MOOSE) Checklist [22]. Articles screening was flowcharted using the Preferred Reporting Items for Systematic Reviews (PRISMA) updated criteria protocol [23]. This study protocol was not registered with the PROSPERO international database.

### Search strategy

We searched the literature through two strategies. The first strategy was to search PubMed, Embase, Medline (OVID), Cochrane Library, and Web of Science databases for NoV-outbreak literature containing information on asymptomatic infections published from the time of database establishment to December 31, 2022. We used different search characters and search terms based on the respective search engine features of the aforementioned databases. The following keywords were searched: “norovirus*,” “norwalk,” “asymptom*,” “diarrh*,” “gastroenter*,” “cluster*,” and “outbreak*.” The second strategy was to screen the relevant references cited by these full-text reading articles in the full-text screening stage, as well as the references of the retrieved reviews and critical articles. There were no restrictions on the study language. If the articles were not published in English, we obtained the required data by getting them translated. The detailed search strategy was presented in Supplementary Files.

### Selection criteria

Two independent reviewers (JW and ZG) initially selected articles that met the study requirements based on their titles and abstracts. During the initial screening process, articles with the following conditions were excluded: (1) The outbreak was not caused by NoV but by other caliciviruses. (2) The subjects were not humans. (3) The subjects were special populations such as HIV-infected individuals. (4) The pathogens were not detected by PCR-based diagnostics methods. (5) The subjects were infected with NoV by human intervention rather than natural infection, such as volunteer challenge studies. (6) The articles were opinion articles and editorial articles.

We then read the full text of the remaining articles in detail and screened for articles that met the research requirements. At this stage, we excluded the articles with the following characteristics: (1) Asymptomatic individuals were reported but not tested. (2) NoV was not detected by PCR-based methods, but by other methods such as serum antibody detection and electron microscopy detection. (3) Prevalence of asymptomatic NoV infection was not reported or could not be calculated. (4) Different articles shared the same data, and only the literature with the complete data was retained. If the two independent reviewers (JW and ZG) disagreed on the above included articles, a third independent reviewer (HZ) took the final decision.

### Data extraction

Our primary summary data included the number of positive asymptomatic subjects along with the total number of asymptomatic individuals tested, i.e., the positive rate. If an article had data on the positive rate, two independent reviewers (JW and ZG) separately extracted the following information from the article: author, publication year, country, the total number of asymptomatic subjects, the number of positive asymptomatic individuals, the positive rate, outbreak setting, outbreak date, transmission route, subjects’ occupations, subjects’ age, sample types, NoV genotypes, and clear definition of the “asymptomatic” individual or the “symptomatic” case. The information about the per capita national income for each country was taken from the World Bank website [24]. Then the two independent reviewers (JW and ZG) entered these data into Epidata 3.0 database and checked the consistency. If there was disagreement, a third independent reviewer (HZ) made the final decision.

Asymptomatic samples were collected from apparently healthy individuals who had a common history of exposure to NoV with the cases in an outbreak. NoV outbreaks have been reported from nursing homes, infant day care centers, kindergartens, schools, catering services, exhibitions, factories, hospitals, and cruise ships. We grouped the above places into four categories: schools and other training institutions, medical institutions, nursing homes, and catering places (including two food-borne outbreaks that occurred on two cruise ships). As the age range of asymptomatic infected individuals in the included studies varied and most did not report specific ages, we did not group them by age. However, we did stratify studies based on whether the study topic was children or adults. For similar reasons, we divided our subjects’ occupations into food handlers and others. We then divided the studies into two groups based on whether they reported a clear case or asymptomatic individual definition.

### Quality assessment

Study quality was assessed according to the following criteria [25]: (1) time, location, and population of the outbreak were clearly described; (2) pathogen testing, health investigations, and interventions were clearly described; (3) investigation of risk factors for the outbreak was clearly described; (4) scientific statistical tests were carried out; and (5) used randomization, stratification, and/or matching to control for bias or discuss any potential confounders. One point was added for each “yes” answer and no point was given for each “no” answer. The maximum score was 5. Studies with a score of 3–5 were considered of high quality and those with a score of 0 were considered of low quality and excluded from the analysis.

### Statistical analysis

We aimed to assess the prevalence of asymptomatic norovirus infection in outbreaks worldwide. For this purpose, we determined the prevalence of asymptomatic NoV infection in an outbreak by dividing the number of subjects without AGE symptoms who tested positive for NoV in stool or other samples by a PCR-based diagnostic method with the total number of asymptomatic individuals tested. An asymptomatic individual in our study should meet one of the following three criteria: (1) The individual met the definition of asymptomatic individuals in the included articles; (2) The individual did not meet the definition of symptomatic cases but had a common history of NoV exposure with symptomatic cases in the included articles; (3) The individual was explicitly mentioned as asymptomatic when there was no cases or asymptomatic individuals definition in the included articles.

We used I square (I^2^) to test significance for heterogeneity. Meta-regression was used to examine the effect of subgroup variables on heterogeneity. R^2^-adjust was used as the percentage of heterogeneity explained by adding variables to the meta-regression model compared to the “null” model. In other words, percentage of heterogeneity was explained using subgroup variables. A random-effects model was used to estimate the asymptomatic prevalence in the overall and subgroup populations because of the expected heterogeneity. For better statistical results, the original proportions were first log-transformed so that they were closer to normal distribution [26]. When the number of NoV-positive individuals was 0, a value of 1/2 was added for the calculation [15, 27]. Differences in asymptomatic prevalence between subgroups were analyzed using the Wilcoxon signed-rank test and Kruskal–Wallis H test, as the data were not normally distributed [25, 27]. Publication bias was assessed by funnel plot and Peter’s test. Sensitivity analysis was carried out by omitting one study at a time. All analyses and mapping were run using R software along with the metafor package and the ArcGIS10.8 (for world map). P-values < 0.05 were considered statistically significant.

## Results

### Literature search and study characteristics

**Fig. 1:**
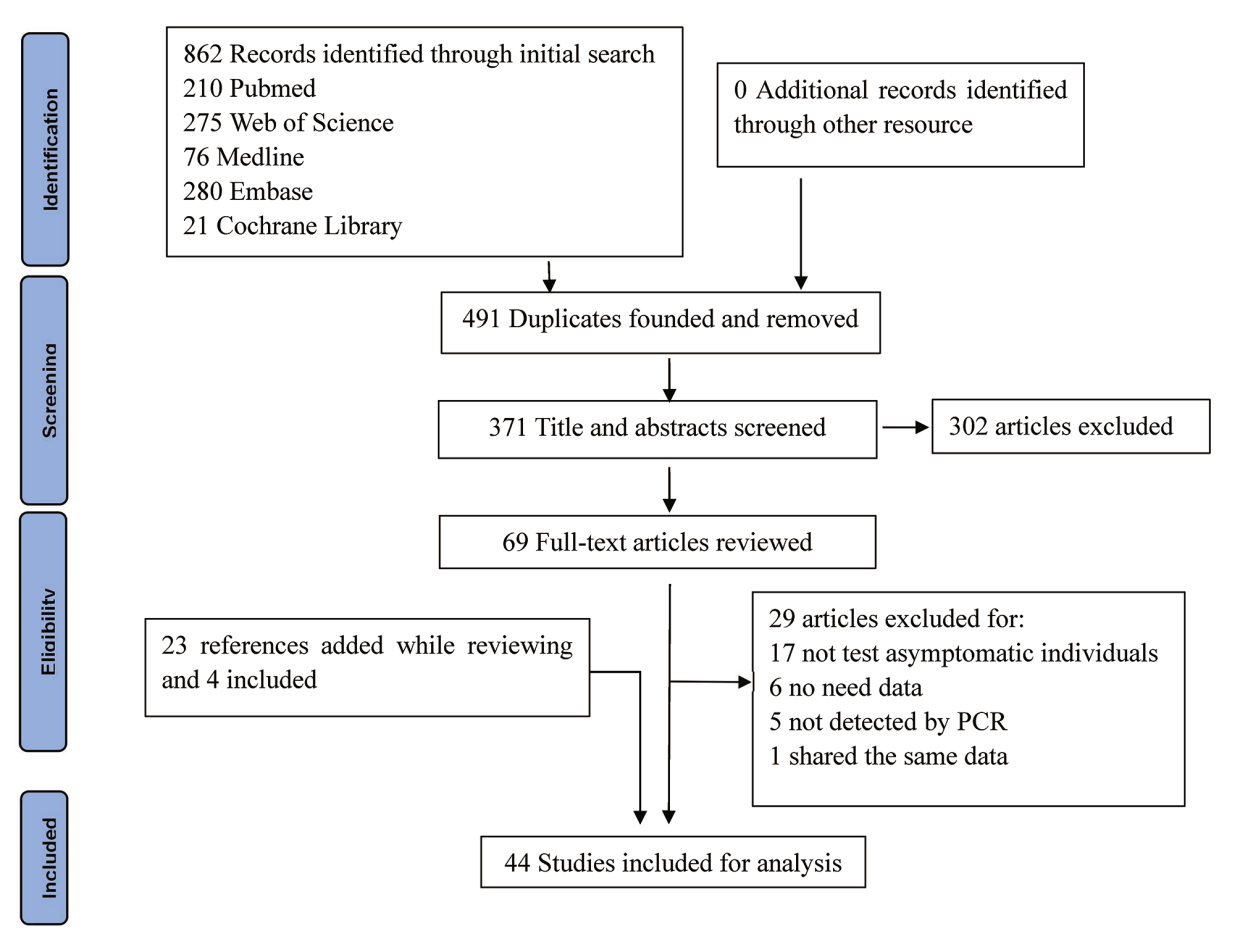
Flow diagram of studies selection

We identified 862 studies through an initial search. Of these, 491 were excluded because of repetition. The remaining 371 were reviewed using their titles and abstracts, and 302 that did not meet the inclusion criteria were excluded. Then we assessed the eligibility of the remaining 69 full-text articles. In this process, we screened the references cited by these articles, as well as the references for reviews and critical articles, adding a total of 23 articles. Of these 23 references, four, including one with mixed infections, were considered for the present study, while the remaining 19 references were either included in the 69 full-text articles or did not meet the inclusion criteria. Finally, 44 articles, 3 of which had mixed infections, were included for the analysis, with a total of 8,115 asymptomatic individuals. Because 10 studies reported outcomes in more than one subgroup, the number of studies in some particular subgroups used for the analysis would exceed the total number of studies included. Figure 1 is the flowchart of articles selection for this study. Table 1 shows the baseline characteristics and quality-assessment results of the studies included in the meta-analysis.

**Table 1:** Summary of characteristics of the studies included in the systematic review

Study	Country	Total number	Positive number	Quality	Outbreak setting	Outbreak season	National income	Subjects’ career	Subjects’ group	Transmission route	Sample types	NoV genotypes	Case definition
Misumi et al. 2021 [1]	Japan	3249	667	2	5	1, 2, 3, 4	High	Food handlers	Adults	4	Fecal	GI + GII	N
Spano et al. 2021 [2]	Brazil	6	3	3	1	2	Mid	Others	Adults	4	Fecal	GII	N
Li et al. 2021 [3]	China	2	1	5	1	1	Upper-mid	Food handlers	Adults	1	Fecal	GII	Y
Wu et al. 2021 [4]	China	11	2	5	1	4	Upper-mid	Food handlers	Adults	1	Fecal	GII	Y
Yu et al. 2021 [5]	China	209	30	3	1	5	Upper-mid	Food handlers, others	All	3	Mixed	GII	Y
Zhang et al. 2020 [6]	China	13	5	4	2	4	Upper-mid	Others	Adults	3	Fecal	GII	Y
Parron et al. 2020 [7]	Spain	415	125	2	5	5	High	Others	All	4	Fecal	GI + GII	Y
Lu et al. 2020 [8]	China	169	10	5	1	1	Upper-mid	Food handlers	Adults	1	Anal swabs	GII	Y
Yang et al. 2019 [9]	China	109	16	2	3	1	Upper-mid	Food handlers	Adults	1	Anal swabs	GII	Y
Wu et al. 2019 [10]	China	377	64	2	1, 2, 3, 4	5	Upper-mid	Food handlers, others	Adults	4	Mixed	GII	Y
Qi et al. 2018 [11]	China	28	3	5	3	1	Upper-mid	Food handlers	Adults	1	Anal swabs	GII	Y
Qian et al. 2018 [12]	China	51	15	3	2	1, 4	Upper-mid	Others	Adults	3	Anal swabs	GII	Y
Shang et al. 2017 [13]	China	59	3	5	1	1	Upper-mid	Food handlers	Adults	2	Anal swabs	GII	Y
He et al. 2016 [14 ]	China	86	14	2	4, 1	4	Upper-mid	Food handlers, others	Adults	3	Fecal	GII	Y
Chen et al. 2016 [15]	China	8	2	5	3	4	Upper-mid	Food handlers	Adults	1	Fecal	GI	Y
Costantini et al. 2016 [16]	America	34	4	1	4	1, 2, 3, 4	High	Others	Adults	4	Fecal	GI, GII	N
Godoy et al. 2016 [17]	Spain	3	2	5	1	4	High	Food handlers	Adults	1	Fecal	GII	Y
Sabrià et al. 2016 [18]	Spain	188	101	2	5	5	High	Others	Adults	4	Fecal	GI + GII	Y
Zheng et al. 2015 [19]	China	76	13	3	4	4	Upper-mid	Others	Adults	3	Anal swabs	GII	Y
Lin et al. 2015 [20]	Austria	3	3	5	1	4	High	Food handlers	Adults	1	Fecal	GII	Y
Wang et al. 2015 [21]	China	31	15	4	3	1	Upper-mid	Others	Adults	2	Anal swabs	GI + GII	Y
Guo et al. 2014 [22]	China	5	2	5	1	4	Upper-mid	Food handlers	All	1	Anal swabs	GII	Y
Xue et al. 2014 [23]	China	44	9	5	1	4	Upper-mid	Food handlers	Adults	1	Anal swabs	GII	Y
Cai et al. 2013 [24]	China	27	8	5	1	4	Upper-mid	Food handlers	Adults	1	Anal swabs	GII	Y
Lai et al. 2013 [25]	China	23	3	3	4	4	Upper-mid	Others	Adults	2	Fecal	GII	Y
Thornley et al. 2013 [26]	New Zealand	8	0	5	1	1	High	Food handlers	Adults	1	Fecal	GII	Y
Feng et al. 2013 [27]	China	26	2	5	1	4	Upper-mid	Food handlers	Adults	1	Anal swabs	GII	Y
Sukhrie et al. 2012 [28]	Netherlands	215	20	1	2	1, 3, 4	High	Others	Adults	4	Fecal	GII	N
Nicolay et al. 2011 [29]	Ireland	5	3	5	3	1	High	Food handlers	Adults	1	Fecal	GII	Y
Schmid et al. 2011 [30]	Austria	20	5	5	2	1	High	Food handlers	Adults	1	Fecal	GII	Y
Yang et al. 2010 [31]	China	249	40	4	4	4	Upper-mid	Others	Adults	3	Fecal	GII	N
Barrabeig et al. 2010 [32]	Spain	2	1	5	1	2	High	Food handlers	Adults	1	Fecal	GII	Y
Yu et al. 2010 [33]	Korea	11	2	5	1	2	High	Food handlers	Adults	1	Fecal	GII	Y
Iizuka et al. 2010 [34]	Japan	11	0	4	4	2	High	Food handlers	Adults	1	Fecal	GII	Y
Kimura et al. 2010 [35]	Japan	85	6	5	3	4	High	Others	Adults	3	Fecal	GII	Y
Medici et al. 2009 [36]	Italy	8	4	5	4	4	High	Others	Adults	1	Fecal	GII	Y
Ozawa et al. 2007 [37]	Japan	1845	133	3	1, 2, 3, 4	1, 3, 4	High	Food handlers	Adults	4	Fecal	GI, GII	Y
Mori et al. 2005 [38]	Japan	256	42	2	2	1, 2, 3, 4	High	Food handlers, others	All	1, 3	Fecal	Unknow	Y
Iizuka et al. 2005 [39]	Japan	12	0	2	1	4	High	Others	Adults	3	Fecal	GII	Y
Gal.limore et al. 2004 [40]	United Kingdom	99	27	2	2	2	High	Others	Adults, children	4	Fecal	GII	N
Hoebe et al. 2004 [41]	Netherlands	16	6	5	1	2	High	Others	Children	2	Fecal	Unknow	Y
Gotz et al. 2002 [42]	Sweden	2	1	5	1	1	High	Food handlers	Adults	1	Fecal	GII	Y
Green et al. 1998 [43]	United Kingdom	16	2	3	2	2	High	Others	Adults	3	Mixed	GII	N
Parashar et al. 1998 [44]	America	3	1	5	1	1	High	Food handlers	Adults	1	Fecal	GII	Y

[ ]: included references number in the section of Included Articles in Additional file 1

Total number: the number of asymptomatic individuals tested in every study

Outbreak setting: (1) Schools and other training institutions; (2) Medical institutions; (3) Catering places; (4) Nursing homes; (5) Unknown

Outbreak season: (1) Spring; (2) Summer; (3) Autumn; (4) Winter; (5) Unknown

Transmission route: (1) Food-borne; (2) Water-borne; (3) Human to human; (4) Unknown

NoV genotype: GI + GII: Prevalence of GI and GII genotypes could not be calculated separately; GI, GII: Prevalence of GI and GII genotypes could be calculated separately

Case definition: Y: With a clear case or asymptomatic individual definition; N: Without a clear case and asymptomatic individual definition

**Fig. 2:**
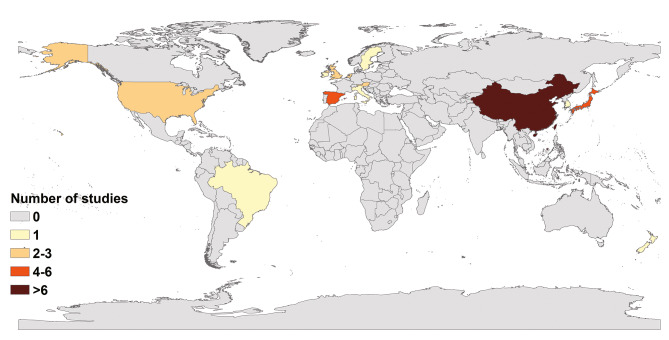
Studies distribution by countries

The 44 articles included in our study were from 13 countries; only two were from the Southern Hemisphere (Fig. 2). The maximum number of studies were from China (20), followed by Japan (6), Spain (4), United States (2), United Kingdom (2), the Netherlands (2), and Austria (2). Only one study was from other countries each such as Ireland, South Korea, Sweden, New Zealand, Italy and Brazil. The majority of the studies were conducted on adults (39). Four of these studies were on the whole population and only one specifically on the prevalence of non-gastrointestinal infectious diseases in children, although it did not mention their age. In addition, only one study was conducted on young children aged 9.2 ± 1.5 years. The outbreak settings comprised schools and other training institutions in 23 articles, catering places in 11 articles, medical institutions in 9 articles, and nursing homes in another 9 articles. Three articles did not report outbreaks settings in detail. In studies with NoV genotype information, 33.36% (472/1415) of positive asymptomatic individuals had GII, 0.92% (13/1415) had GI, 1.55% (22/1415) did not report the genotype (here, 1415 refers to the number of asymptomatic individuals with pathogens); and the rest (64.17%, 908/1415) reported genotypes, however, it was difficult to pinpoint the individual. In addition, 79.54% (35/44) of included articles reported only NoV GII, 2.27% (1/44) reported only NoV GI, 13.64% (6/44) reported both NoV GII and NoV GI, and 4.55% (2/44) reported no NoV genotypes. Among these studies, 37 clearly defined NoV cases or “asymptomatic” individuals, whereas seven studies mentioned “asymptomatic” but did not define them.

### Meta-analysis of total asymptomatic prevalence

**Fig. 3:**
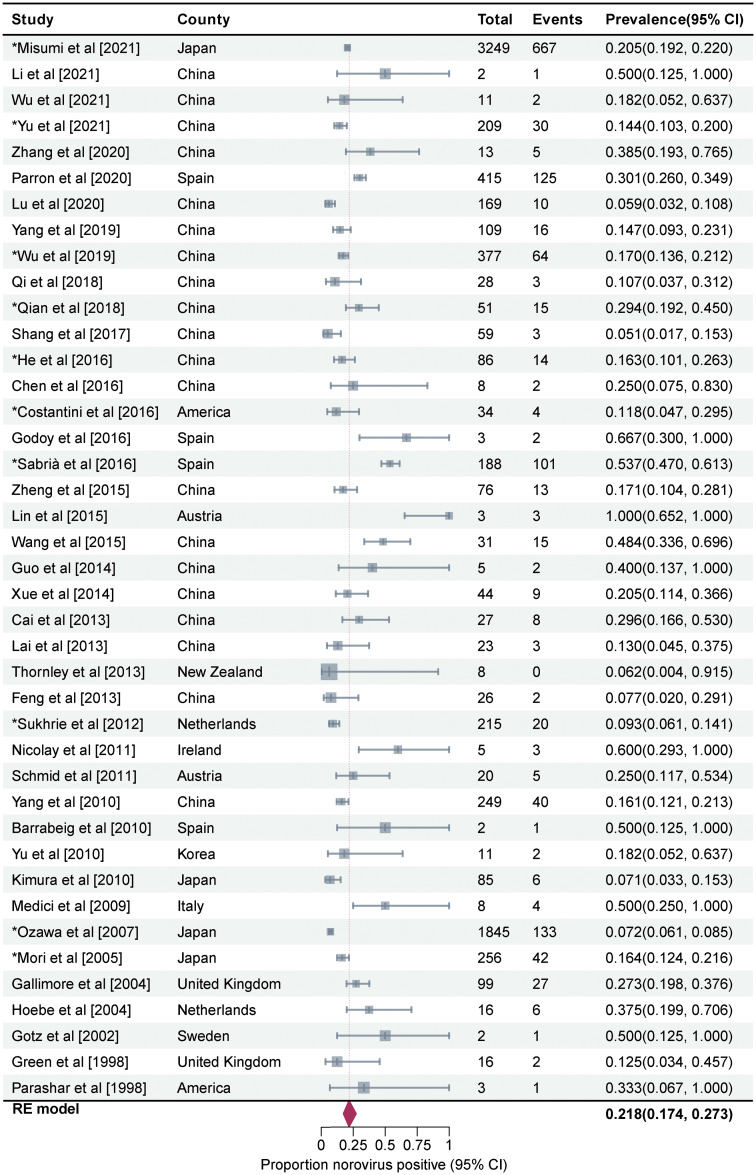
Forest graph. Results of 44 studies estimating the prevalence of asymptomatic NoV infection in the outbreaks (I^2^ = 92%, τ^2^ = 0.4021, P < 0.01 test for heterogeneity). Events: Number of NoV-positive asymptomatic individuals. Total: Number of asymptomatic individuals whose samples were detected. *Studies with prevalence were calculated in *N* outbreaks (N > 1)

Prevalence of asymptomatic NoV infection in outbreaks was estimated to be 21.8% (95%CI, 17.4–27.3, I^2^ = 92%, τ^2^ = 0.4021, P < 0.01 test for heterogeneity) by using a random-effects model for the 44 articles included in this study (Fig. 3). Three studies on mixed infections were excluded then. The remaining 41 studies reported a prevalence rate of 21.8% for asymptomatic NoV infection in outbreaks (95%CI, 17.4–27.3, I^2^ = 93%, τ^2^ = 0.3990, P < 0.001 test for heterogeneity). No statistical difference was observed between the total prevalence of 41 and 44 studies. We performed a meta-regression analysis with a single covariate for the following 10 factors to determine the source of heterogeneity: geographical area, outbreak settings, outbreak seasons, sample types, genotypes, transmission routes, subjects’ occupations, subjects’ age, per capita national income, and presence or absence of a well-defined case and/or asymptomatic individual. Only the meta-regression analysis results for the geographical area were statistically significant (P = 0.012). The between-study variance decreased from 0.3369 to 0.2925, suggesting that it could explain 13.18% of the heterogeneity.

### Meta-analysis of subgroup asymptomatic prevalence

**Fig. 4:**
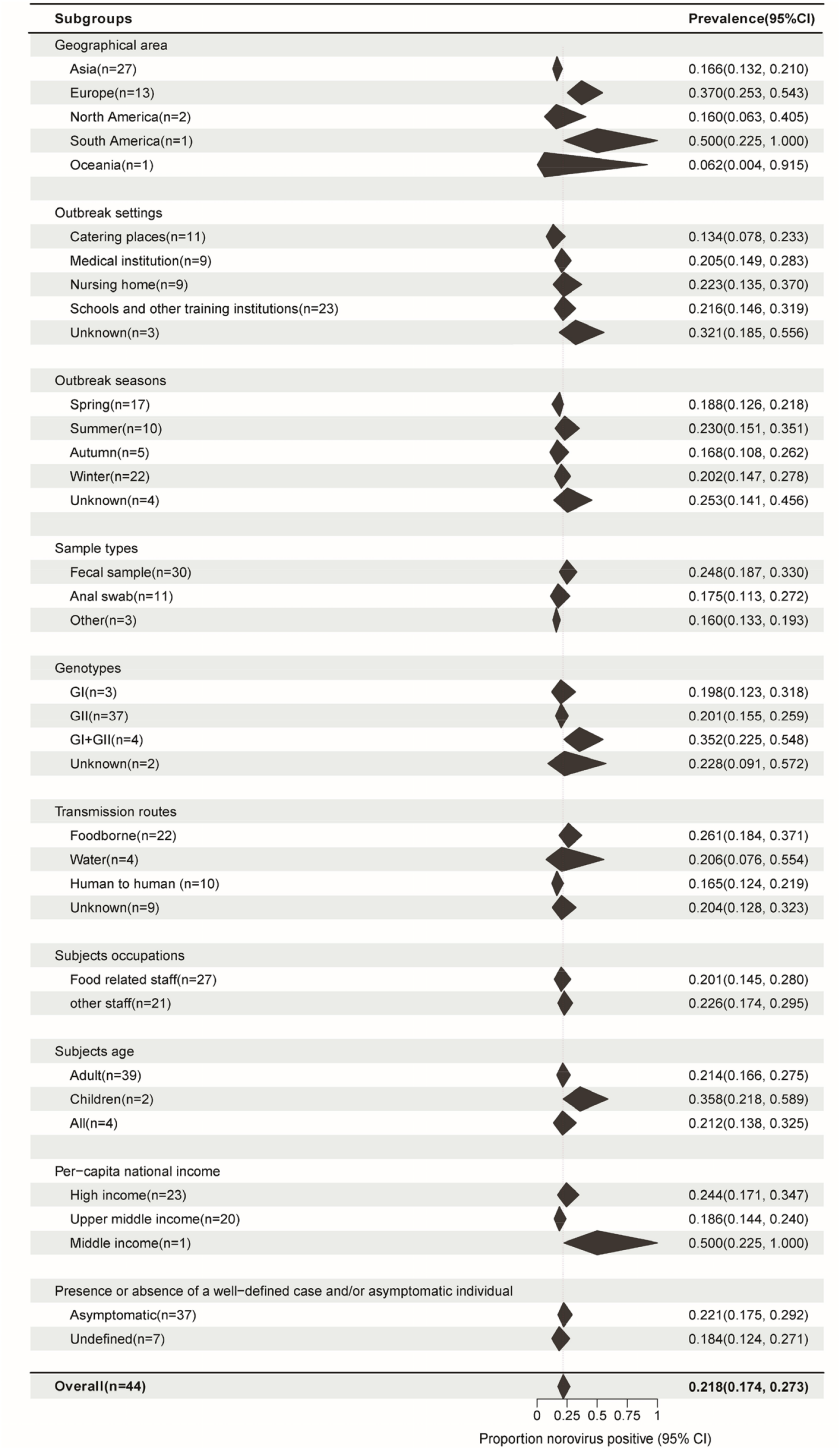
Subgroup pooled prevalence results. *N*: Number of studies. Black polygon: Estimated prevalence for each subgroup. Polygon width: Confidence interval of the pooled estimate. Because some studies involved information from multiple outbreaks, the number of studies from certain specific subgroups used in the analysis was not added to the total. Overall summary estimates were added to allow for comparison via the polygons with red dashed lines. For all subgroups, *P* values for heterogeneity tests P < 0.01. There are various sources of heterogeneity in prevalence studies. Subgroups divided by each factor still have significant heterogeneity

The pooled asymptomatic prevalence results for the subgroups are shown in Fig. 4. To determine the source of heterogeneity in subgroup analysis, it is necessary that the within-group heterogeneity should not be significant in any of the groups. However, the results of subgroup analysis showed that some groups had significant heterogeneity for each grouping factor (P < 0.05). That is, the source of heterogeneity could not be determined for the above 10 grouping factors.

**Fig. 5:**
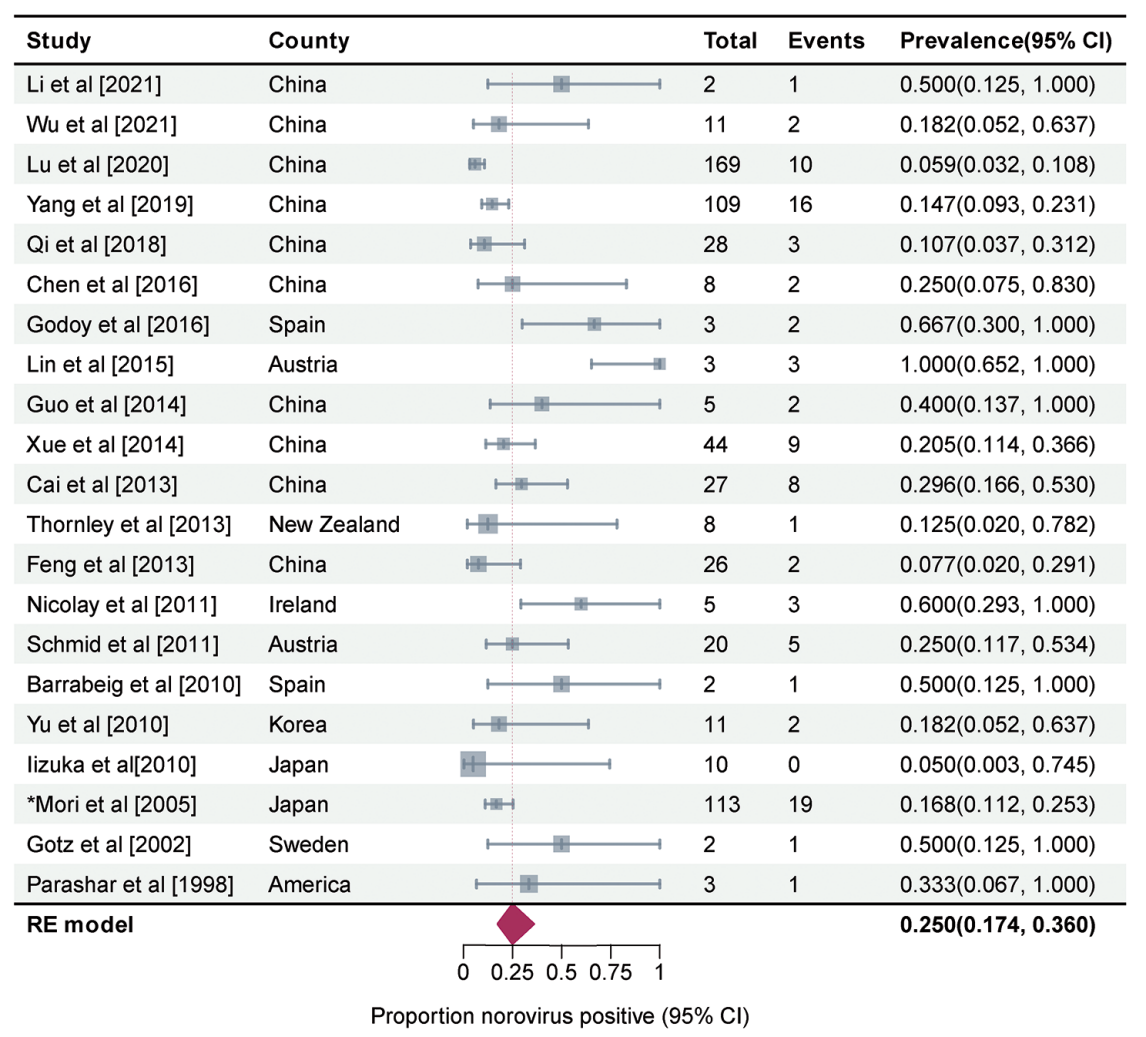
Forest graph: Meta-analysis of 21 studies estimating the prevalence of asymptomatic food handlers in foodborne outbreaks (I^2^ = 79%, τ^2^ = 0.4500, P < 0.01 test for heterogeneity). Events: Number of NoV-positive asymptomatic individuals. Total: Number of asymptomatic individuals whose samples were detected. *Studies with prevalence were calculated in *N* outbreaks (N > 1)

**Fig. 6:**
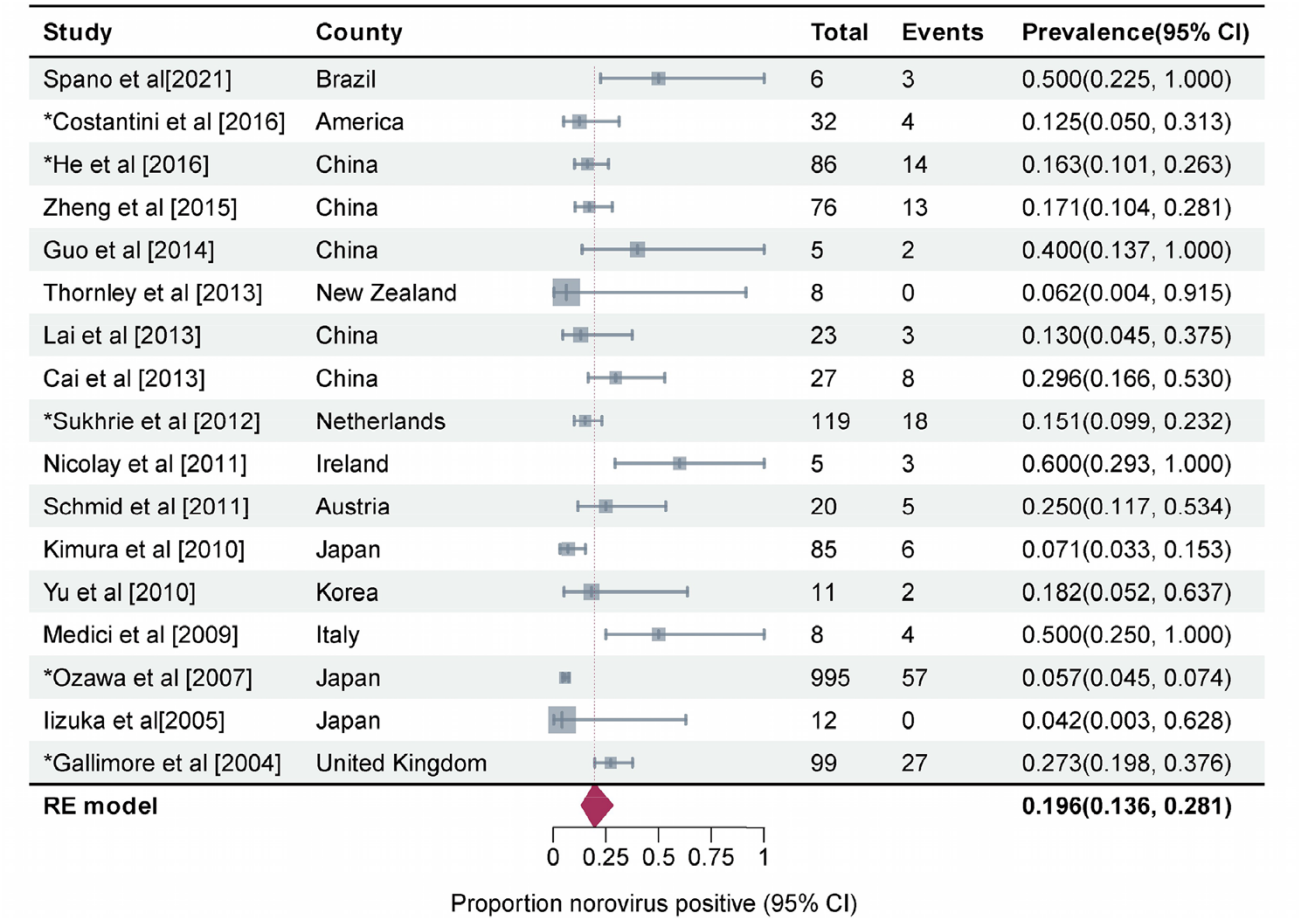
Forest graph: Meta-analysis of 17 studies estimating the prevalence of asymptomatic NoV GII.4 infection in outbreaks (I^2^ = 87%, τ^2^ = 0.3985, P < 0.01 test for heterogeneity). Events: Number of NoV-positive asymptomatic individuals. Total: Number of asymptomatic individuals whose samples were detected. *Studies with prevalence were calculated in *N* outbreaks (N > 1)

If we consider the geographic region, the prevalence was high in Europe (37.0%, 95%CI, 25.3–54.3) and low in East Asia (16.6%, 95%CI, 13.2–21.0) (*P* = 0.001). Among the outbreak settings, the prevalence rates in schools and other training institutions, medical institutions, nursing homes, and catering places were 21.6% (95%CI, 14.6–31.9), 20.5% (95%CI, 14.9–28.3), 22.3% (95%CI, 13.5–37.0), and 13.4% (95%CI, 7.8–23.3), respectively. However, the seasonal characteristics of the prevalence were not obvious; the highest prevalence was noted in the summer season (23.0%, 95%CI, 15.1–35.1), followed by that in the winter (20.2%, 95%CI, 14.7–27.8), spring (18.8%, 95%CI, 12.6–21.8), and autumn (16.8%, 95%CI, 10.8–26.2). The prevalence was the highest in foodborne outbreaks ((26.1%, 95%CI, 18.4–37.1), it was 25.0% (95%CI, 17.4–36.0) among food handlers (Fig. 5)), followed by waterborne outbreaks (20.6%, 95%CI, 7.6–55.4) and human-to-human outbreaks (16.5%, 95%CI, 12.4–21.9). Studies that collected only stool and anal swab samples reported prevalence rates of 24.8% (95%CI, 18.7–33.0) and 17.5% (95%CI, 11.3–27.2), respectively, while those that collected different specimens such as feces, vomitus, anal swabs, and throat swabs had a prevalence rate of 16.0% (95%CI, 13.3–19.3). The mixed infection prevalence of GI and GII NoV was the highest (35.2%, 95%CI, 22.5–54.8), followed by that of GII (20.1%, 95%CI, 15.5–25.9) and GI (19.8%, 95%CI, 12.3–31.8) alone. For NoV GII.4, the prevalence was estimated to be 19.6% (95%CI, 13.6–28.1) (Fig. 6), which was similar to that for other GII subtypes (18.2%, 95%CI, 12.4–26.8) (Supplementary Figure. S1) in outbreaks. The estimated prevalence for food handlers was 20.1% (95%CI, 14.5–28.0), which was similar to that for other occupational populations (22.6%, 95%CI, 17.4–29.5). The prevalence was higher in children (35.8%, 95%CI, 21.8–58.9) than it was in adults (21.4%, 95%CI, 16.6–27.5), as well as higher in high-income countries (24.4%, 95%CI, 17.1–34.7) than in upper-middle-income countries (18.6%, 95%CI, 14.4–24.0). The prevalence in studies that provided a clear definition of the case or asymptomatic individual was 22.1% (95%CI, 17.5–29.2), which was not significantly different from that in studies that did not provide such a clear definition (18.4%, 95%CI, 12.4–27.1). Except for the geographic region, the subgroup analysis of the other nine factors did not show any significant difference in prevalence among the groups.

### Sensitivity analysis and publication bias

Statistically, the funnel plot indicates that the scatter plots in the image are basically symmetric (Supplementary Figure. S2). According to the funnel plot and Peter’s test results (*P* = 0.251), there was no significant publication bias. Sensitivity analysis was carried out by omitting one study at a time, and the results are very stable (Supplementary Figure. S3).

## Discussion

NoV could be detected in asymptomatic individuals. Similar to symptomatic cases, asymptomatic individuals with NoV can also continuously shed virus with similar shedding patterns (i.e., viral load and shedding duration) [8, 9]. This means that asymptomatic individuals can also cause NoV outbreaks [28, 29]. Hence, ignoring asymptomatic individuals during an outbreak may prolong its duration and increase its size. Therefore, it is necessary to analyze the asymptomatic prevalence of NoV in outbreaks to obtain some reference for their emergency disposal measures and prevention and control strategies.

According to the 44 studies that we used for our analysis, the prevalence of asymptomatic NoV infection in outbreaks was 21.8%, much higher than that found during routine surveillance (7–8%) [15, 30], but still lower than that reported in volunteer infection studies (32%) [31]. This may be due to the gradual decrease in the likelihood and dose of asymptomatic subjects exposed to NoV from volunteer challenge studies to outbreaks to routine surveillance.In general, asymptomatic subjects are inevitably exposed to NoV in volunteer challenge studies, and the exposure dose is higher than the pathogenic dose [31]. Close contacts in an outbreak are more likely to be exposed to NoV than people in their daily lives, but the dose of the virus exposed varies and not everyone will be exposed. The likelihood and exposure dose of NoV is the lowest for healthy individuals in their daily lives. Another meta-analysis involving 15 studies on norovirus outbreaks reported a lower asymptomatic prevalence (18%) [15]. However, the above mentioned meta-analysis mainly estimated the asymptomatic prevalence of NoV among populations in routine surveillance (7%, 95% CI, 6–9). This could be because we included a large number of studies (44) from more countries (13) over a longer period of time, which collected data only between 2004 and 2016. A large global meta-analysis estimated the prevalence of NoV to be approximately 18% in patients with AGE, which may increase in outbreaks (45%) [2, 30]. The high prevalence of both symptomatic and asymptomatic NoV infections suggests that this pathogen presents a high hazard and heavy burden. Hence, targeted control measures are needed to halt the scale and duration of NoV outbreaks.

In practice, the prevalence of asymptomatic NoV infection in an outbreak may be affected by many factors such as geographic distribution, outbreak settings, NoV genotypes, and population characteristics. Meta-regression analysis suggests that the source of heterogeneity in asymptomatic prevalence could be related to the geographic location of the outbreaks. The asymptomatic prevalence is much higher in Europe than it is in other continents, which may be due to many factors, such as more outbreaks reported in semi-enclosed and enclosed places, more reports of foodborne outbreaks and more collection of stool samples. High-income countries have a slightly higher asymptomatic prevalence than upper-middle-income countries; this difference is not significant. The two groups of countries have similar levels of water sanitation, hygiene, and sanitation; the small difference might be due to different levels of aging of the population. However, a recent meta-analysis of the global prevalence of NoV did not find any significant association between the national income level and its prevalence [3].

Asymptomatic prevalence is the highest in nursing homes, followed by schools and other training institutions, medical institutions, and catering venues, possibly in part owing to differences in the frequency and timing of exposure between cases and healthy individuals in these outbreak settings [2, 3]. The professional activities of medical staff and full-time faculty will lead to closer and longer-term contacts with the patients and students they attend, making them more susceptible to NoV infections. In contrast, kitchen workers, who are in less direct contact with clients, have a lower risk of asymptomatic infection. Parron et al. reported that the greatest risk of NoV outbreaks was through direct human-to-human transmission [32]. The prevalence of asymptomatic NoV infection was the highest in foodborne outbreaks, followed by waterborne and human-to-human outbreaks. This may be related to the likelihood and dose of subjects exposure to NoV over a short period of time gradually decrease in general, from food-borne outbreaks to water-borne outbreaks to human-to-human outbreaks [33, 34]. In human-to-human outbreaks, some close contacts may not even be exposed to the virus.

NoV is also known as the “winter vomiting disease” and may cause sporadic cases as well as outbreaks in the winter [35, 36]. Surprisingly, our study did not show any significant seasonal differences in the asymptomatic prevalence of NoV in outbreaks. This is most likely due to the high population density in these outbreak settings [37]. For the same reason, there was no significant difference in the asymptomatic prevalence of NoV GII.4 and other GII subtypes in outbreaks. The same was true for food handlers and other occupational groups. These results are different from those obtained through routine surveillance, which reported a lower asymptomatic prevalence (3%) for food workers than that for the general population (7%) [26]. The higher asymptomatic prevalence in children suggests that it may be related to their immunity, because children may be more resistant to the disease than adults are [4, 38]. Previous study has found that the NoV prevalence peaked between 6 and 23 months in young children, afterwards, it fell into trough which around 4 to 14 years of age, before the second peak located in adults and elderly [4]. To some extent, this supports that the immunity of individuals infected with NoV could last longer (4 years and more) rather than short-term (6 months to 2 years) [38]. NoV-detection rates were higher for stool samples of asymptomatic individuals than they were for anal swabs and mixed samples. This is mainly because NoV diarrhea is a gastrointestinal infectious disease and the amount of virus in feces is higher [39, 40]. The CDC also recommends using a whole stool sample to diagnose NoV gastroenteritis by real-time PCR when conditions permit [40]. In our study, the asymptomatic prevalence of NoV GII (20.1%) was similar to that of GI (19.8%), the proportion of asymptomatic individuals involved in the former (33.36%) was much higher than that of in the latter (0.92%), and the former (93.18%) was reported much more frequently than the latter (15.91%) in the included articles. Thus NoV GII is the dominant genotype associated with asymptomatic infection in outbreaks.

Our study has several limitations as well. First, because of the lack of detailed data, we could not accurately distinguish and calculate the asymptomatic prevalence in older adults and children in outbreaks. And the existing asymptomatic prevalence in children based on two studies should be treated with caution. Second, the definition of asymptomatic individual varies from study to study. In well-defined articles, the absence of at least two major symptoms (vomiting and diarrhea) was required to be considered an asymptomatic individual. Some articles also required the absence of fever, abdominal pain, and nausea. Third, host genetic factors were not taken into account, nor were they reported in the included literature. NoV could easily bind to the histo-blood group antigens (HBGA) expressed by the *FUT2* gene [41]. A systematic review of the association between HBGA and NoV susceptibility showed that individuals carrying the *FUT2* gene were 2.2–9.9 times more likely to be infected than non-carriers [42]. Fourth, the studies did not report diarrhea-free periods in asymptomatic individuals before the outbreak or whether asymptomatic individuals developed clinical symptoms within a maximum incubation period after specimen collection. Therefore, we may have overestimated the asymptomatic prevalence of NoV in outbreaks. Fifth, geographic and country representation is incomplete. There is a lack of data from studies in Africa, Central Asia, and other regions, as well as from low- and lower-middle-income countries. Sixth, sampling methods of asymptomatic individuals tested were not reported in all but two of the articles. Seventh, multi-factors meta-regression analysis was not performed because many articles reported outcomes in more than one subgroup. Therefore, we should be cautious about the heterogeneity of geographical distribution on research results. Finally, the I^2^-test for model heterogeneity is highly significant, possibly because the included articles were limited. Many factors that might explain the source of heterogeneity, such as age, genotypes, and transmission routes, do not show heterogeneity themselves. In addition, other factors not considered may have significant impacts on the prevalence of asymptomatic infection. Despite these limitations, our study still provides detailed information on the prevalence of asymptomatic NoV infection in outbreaks, which can be useful for emergency management of outbreaks.

## Conclusions

Prevalence of asymptomatic NoV infection estimated by meta-analysis in the context of outbreaks exposure is as high as 21.8%, with genome II dominating. Such a high prevalence indicates that asymptomatic individuals should not be ignored and that they could play an important role in NoV transmission. This knowledge may have a significant impact on the development of containment strategies for NoV outbreaks.

### Electronic supplementary material

Below is the link to the electronic supplementary material.

Supplementary Material 1

## Data Availability

The datasets supporting the conclusions of this article are available through the published studies listed in Table 1 and Additional file 1.

## Abbreviations

NoV: Norovirus
AGE: Acute gastroenteritis
HBGA: Histo-blood group antigens
