# Supplementary material for: Global prevalence of asymptomatic norovirus infection in outbreaks: a systematic review and meta-analysis
Source: BMC Infect Dis. 2023 Sep 12;23:595. doi: 10.1186/s12879-023-08519-y (PMC10496210; doi:10.1186/s12879-023-08519-y)
Supplement: Supplementary file 1 — Supplementary Material 1 [file 12879_2023_8519_MOESM1_ESM.docx]

**Supplemental Materials**

**Global prevalence of asymptomatic norovirus infection in outbreaks: A** **systematic review and** **meta-analysis**

Jun Wang, Zhao Gao, Zu-rong Yang, Kun Liu, Hui Zhang

**Contents**

[Supplemental Table S1: Meta-analyses Of Observational Studies in Epidemiology (MOOSE) Checklist 2](#_Toc130148038)

[Supplemental Table S2: Search strategy for PubMed database 4](#_Toc130148038)

[Supplemental Table S3: Search strategy for Embase database 5](#_Toc130148038)

[Supplemental Table S4: Search strategy for Cochrane Library database 6](#_Toc130148038)

[Supplemental Table S5: Search strategy for Medline (OVID) database 7](#_Toc130148038)

[Supplemental Table S6: Search strategy for Web of Science database 8](#_Toc130148038)

[Supplemental Figure S1: Prevalence of](#_Toc130148040) [norovirus](javascript:;) [genotypes GII without GII.4](#_Toc130148040) 9

[Supplemental Figure S2: Funnel plot for assessment of publication bias 1](#_Toc130148040)0

[Supplemental Figure S3: Sensitivity analysis of meta-analysis 1](#_Toc130148040)1

[Supplemental Figure S4: Subgroup prevalence results of geographic distribution 1](#_Toc130148038)2

[Supplemental Figure S5: Subgroup prevalence results of outbreaks settings 1](#_Toc130148039)3

[Supplemental Figure S6: Subgroup prevalence results of outbreaks seasons 1](#_Toc130148040)4

[Supplemental Figure S7: Subgroup prevalence results of transmission routes 1](#_Toc130148040)5

[Supplemental Figure S8: Subgroup prevalence results of samples types 1](#_Toc130148040)6

[Supplemental Figure S9: Subgroup prevalence results of](#_Toc130148040) [norovirus](javascript:;) [genotypes 17](#_Toc130148040)

[Supplemental Figure S10: Subgroup prevalence results of subjects' occupations 1](#_Toc130148040)8

[Supplemental Figure S11: Subgroup prevalence results of subjects' age 1](#_Toc130148040)9

[Supplemental Figure S12: Subgroup prevalence results of per capita national income 2](#_Toc130148040)0

[Supplemental Figure S13:](#_Toc130148040) [Subgroup prevalence results of case definition 2](#_Toc130148040)1

[Included Articles 22](#_Toc130148046)

**[Supplemental Table S1:](#_Toc130148037)** [Meta-analyses Of Observational Studies in Epidemiology (MOOSE) Checklist](#_Toc130148037)

| Item No | Recommendation | Reported on Page No |
| --- | --- | --- |
| Reporting of background should include | | |
| 1 | Problem definition | 4-5,9 |
| 2 | Hypothesis statement | 4-5 |
| 3 | Description of study outcome(s) | 5 |
| 4 | Type of exposure or intervention used | 4-5 |
| 5 | Type of study designs used | 4-5 |
| 6 | Study population | 8,9 |
| Reporting of search strategy should include | | |
| 7 | Qualifications of searchers (eg, librarians and investigators) | 6-8 |
| 8 | Search strategy, including time period included in the synthesis and key words | 5-6,  Suppl Table S2-S6 |
| 9 | Effort to include all available studies, including contact with authors | 6 |
| 10 | Databases and registries searched | 6 |
| 11 | Search software used, name and version, including special features used (eg, explosion) | -NA- |
| 12 | Use of hand searching (eg, reference lists of obtained articles) | 6 |
| 13 | List of citations located and those excluded, including justification | 6-7,  Fig 1 |
| 14 | Method of addressing articles published in languages other than English | 5-6 |
| 15 | Method of handling abstracts and unpublished studies | 6-7 |
| 16 | Description of any contact with authors | -NA- |
| Reporting of methods should include | | |
| 17 | Description of relevance or appropriateness of studies assembled for assessing the hypothesis to be tested | 5 |
| 18 | Rationale for the selection and coding of data (eg, sound clinical principles or convenience) | 7-9 |
| 19 | Documentation of how data were classified and coded (eg, multiple raters, blinding and interrater reliability) | 7-9 |
| 20 | Assessment of confounding (eg, comparability of cases and controls in studies where appropriate) | 5,8-9 |
| 21 | Assessment of study quality, including blinding of quality assessors, stratification or regression on possible predictors of study results | 8-9 |
| 22 | Assessment of heterogeneity | 9-10 |
| 23 | Description of statistical methods (eg, complete description of fixed or random effects models, justification of whether the chosen models account for predictors of study results, dose-response models, or cumulative meta-analysis) in sufficient detail to be replicated | 9-10 |
| 24 | Provision of appropriate tables and graphics | Table 1, Fig 1-6 |
| Reporting of results should include | | |
| 25 | Graphic summarizing individual study estimates and overall estimate | Fig 3-6 |
| 26 | Table giving descriptive information for each study included | Table 1 |
| 27 | Results of sensitivity testing (eg, subgroup analysis) | 13-15,  Fig 4, Suppl Fig S2-S3 |
| 28 | Indication of statistical uncertainty of findings | 12-15,  Fig 3-6 |
| Reporting of [discussion](javascript:;) should include | | |
| 29 | Quantitative assessment of bias (eg, [publication](javascript:;) [bias](javascript:;)) | 15,19-20 |
| 30 | Justification for exclusion (eg. Exclusion of non-English-language citations) | -NA- |
| 31 | Assessment of quality of included studies | Table 1 |
| Reporting of conclusions should include | | |
| 32 | Consideration of alternative explanations for [observed](javascript:;) [result](javascript:;)s | 19-20 |
| 33 | Generalization of the conclusions (ie.appropriate for the data presented and within the domain of the literature review) | 20 |
| 34 | Guidelines for future research | 19-20 |
| 35 | Disclosure of funding source | 21 |

*From:* Stroup DF, Berlin JA, Morton SC, Olkin I, Williamson GD, Rennie D, Moher D, Becker BJ, Sipe TA, Thacker SB. Meta-analysis of observational studies in epidemiology: a proposal for reporting. Meta-analysis Of Observational Studies in Epidemiology (MOOSE) group. JAMA. 2000;283(15):2008-12. doi: 10.1001/jama.283.15.2008.

**Supplemental Table S2:** Search strategy for PubMed

| # | Searches | Results |
| --- | --- | --- |
| 1 | "norovirus"[MeSH Terms] | 5,509 |
| 2 | "norovirus*"[All Fields] | 7,204 |
| 3 | "norwalk*"[All Fields] | 2,144 |
| 4 | "norovirus"[MeSH Terms] OR "norovirus*"[All Fields] OR "norwalk*"[All Fields] | 8,974 |
| 5 | "acute*"[All Fields] AND "gastroenter*"[All Fields] | 50,713 |
| 6 | "diarrh*"[MeSH Terms] OR "diarrh*"[All Fields] | 143,258 |
| 7 | "disease outbreaks"[MeSH Terms] OR ("disease*"[All Fields] AND "outbreak*"[All Fields]) | 254,132 |
| 8 | "cluster*"[All Fields] | 518,108 |
| 9 | ("acute*"[All Fields] AND "gastroenter*"[All Fields]) OR "diarrh*"[MeSH Terms] OR "diarrh*"[All Fields] OR "disease outbreaks"[MeSH Terms] OR ("disease*"[All Fields] AND "outbreak*"[All Fields]) OR "cluster*"[All Fields] | 936,398 |
| 10 | "latent*"[All Fields] AND "infect*"[All Fields] | 31,642 |
| 11 | "asymptomatic infections"[MeSH Terms] AND "asymptomat*"[All Fields] | 2,659 |
| 12 | ("norovirus"[MeSH Terms] OR "norovirus*"[All Fields] OR "norwalk*"[All Fields]) AND (("acute*"[All Fields] AND "gastroenter*"[All Fields]) OR "diarrh*"[MeSH Terms] OR "diarrh*"[All Fields] OR "disease outbreaks"[MeSH Terms] OR ("disease*"[All Fields] AND "outbreak*"[All Fields]) OR "cluster*"[All Fields]) AND (("latent*"[All Fields] AND "infect*"[All Fields]) OR "asymptomatic infections"[MeSH Terms] OR "asymptomat*"[All Fields]) | 211 |
| 13 | (("norovirus"[MeSH Terms] OR "norovirus*"[All Fields] OR "norwalk*"[All Fields]) AND (("acute*"[All Fields] AND "gastroenter*"[All Fields]) OR "diarrh*"[MeSH Terms] OR "diarrh*"[All Fields] OR "disease outbreaks"[MeSH Terms] OR ("disease*"[All Fields] AND "outbreak*"[All Fields]) OR "cluster*"[All Fields]) AND (("latent*"[All Fields] AND "infect*"[All Fields]) OR "asymptomatic infections"[MeSH Terms] OR "asymptomat*"[All Fields])) AND (1978/1/1:2022/12/31[pdat]) | 210 |

**Supplemental Table S3:** Search strategy for Embase

| # | Searches | Results |
| --- | --- | --- |
| 1 | norovirus*:ti,ab,kw | 8,158 |
| 2 | norwalk*:ti,ab,kw | 1,486 |
| 3 | gastroenter*:ti,ab,kw | 107,930 |
| 4 | diarrh*:ti,ab,kw | 189,561 |
| 5 | outbreak*:ti,ab,kw | 146,176 |
| 6 | cluster*:ti,ab,kw | 525,404 |
| 7 | latent* AND infect*:ti,ab,kw | 39,300 |
| 8 | asymptom*:ti,ab,kw | 269,684 |
| 9 | #1 OR #2 | 9,329 |
| 10 | #5 OR #6 | 661,944 |
| 11 | #7 OR #8 | 307,187 |
| 12 | #9 AND #10 AND #11 | 132 |
| 13 | #3 OR #4 | 285,436 |
| 14 | #9 AND #11 AND #13 | 252 |
| 15 | (#12 OR #14) AND [01-01-1942]/sd NOT [01-01-2023]/sd | 280 |

**Supplemental Table S4:** Search strategy for Cochrane Library

| # | Searches | Results |
| --- | --- | --- |
| 1 | MeSH descriptor: [Norovirus] explode all trees | 30 |
| 2 | (Noroviruses or Norwalk like Viruses or Norwalk-like Viruses or Round-Structured Viruses, Small or Small Round-Structured Viruses or Small Round Structured Viruses):ti,ab,kw | 138 |
| 3 | (outbreak* or cluster*):ti,ab,kw | 32,587 |
| 4 | (gastroenter*):ti,ab,kw | 6,503 |
| 5 | Infection, Subclinical or Inapparent Infection or Inapparent Infections or Subclinical Infections or Infection, Asymptomatic or Asymptomatic Infection or Infection, Inapparent or Subclinical Infection or Asymptomatic Colonization or Asymptomatic Colonizations or Colonization, Asymptomatic or Presymptomatic Infections or Infection, Presymptomatic or Presymptomatic Infection | 3,439 |
| 6 | #1 or #2 | 138 |
| 7 | #4 and #3 and #6 | 7 |
| 8 | #6 and #3 | 21 |
| 9 | #8 and #5 | 1 |
| 10 | #7 or #8 or #9 | 21 |

**Supplemental Table S5:** Search strategy for Medline (OVID)

| # | Searches | Results |
| --- | --- | --- |
| 1 | (norovirus* or Norwalk*).mp. [mp=title, book title, abstract, original title, name of substance word, subject heading word, floating sub-heading word, keyword heading word, organism supplementary concept word, protocol supplementary concept word, rare disease supplementary concept word, unique identifier, synonyms] | 8,198 |
| 2 | ((gastroenter* or diarrh*) adj3 outbreak*).mp. [mp=title, book title, abstract, original title, name of substance word, subject heading word, floating sub-heading word, keyword heading word, organism supplementary concept word, protocol supplementary concept word, rare disease supplementary concept word, unique identifier, synonyms] | 3,059 |
| 3 | ((norovirus* or Norwalk*) adj3 outbreak*).mp. [mp=title, book title, abstract, original title, name of substance word, subject heading word, floating sub-heading word, keyword heading word, organism supplementary concept word, protocol supplementary concept word, rare disease supplementary concept word, unique identifier, synonyms] | 961 |
| 4 | (latent* infect* or Asymptom*).mp. [mp=title, book title, abstract, original title, name of substance word, subject heading word, floating sub-heading word, keyword heading word, organism supplementary concept word, protocol supplementary concept word, rare disease supplementary concept word, unique identifier, synonyms] | 195,282 |
| 5 | 1 and 2 | 1,120 |
| 6 | 3 and 4 | 52 |
| 7 | 4 and 5 | 47 |
| 8 | 6 or 7 | 77 |
| 9 | Limit 8 to yr=”1860-2022” | 76 |

**Supplemental Table S6:** Search strategy for Web of Science

| # | Searches | Results |
| --- | --- | --- |
| 1 | ALL=(norovirus*) | 8,314 |
| 2 | ALL=(norwalk*) | 6,365 |
| 3 | ALL=(gastroenter*) | 856,300 |
| 4 | ALL=(diarrh*) | 125,603 |
| 5 | ALL=(outbreak*) | 183,469 |
| 6 | ALL=(cluster*) | 1,143,888 |
| 7 | ALL=(latent* infect*) | 32,966 |
| 8 | ALL=(asymptom*) | 158,184 |
| 9 | #1 OR #2 | 12,835 |
| 10 | #5 OR #6 | 1,315,782 |
| 11 | #7 OR #8 | 189,918 |
| 12 | #9 AND #10 AND #11 | 160 |
| 13 | #3 OR #4 | 956,817 |
| 14 | #9 AND #11 AND #13 | 257 |
| 15 | #12 OR #14 Retrieval period: 1980-01-01 to 2022-12-31 | 275 |

**Fig S1.** Forest graph: Prevalence results of [norovirus](javascript:;) genotypes GII without GII.4 (*I^2^* = 87%, *τ^2^* = 0.6218, *P* < 0.01 test for heterogeneity). Events: Number of NoV-positive asymptomatic individuals. Total: Number of asymptomatic individuals whose samples were detected. *Studies with prevalence were calculated in *N* outbreaks (*N* > 1).

**
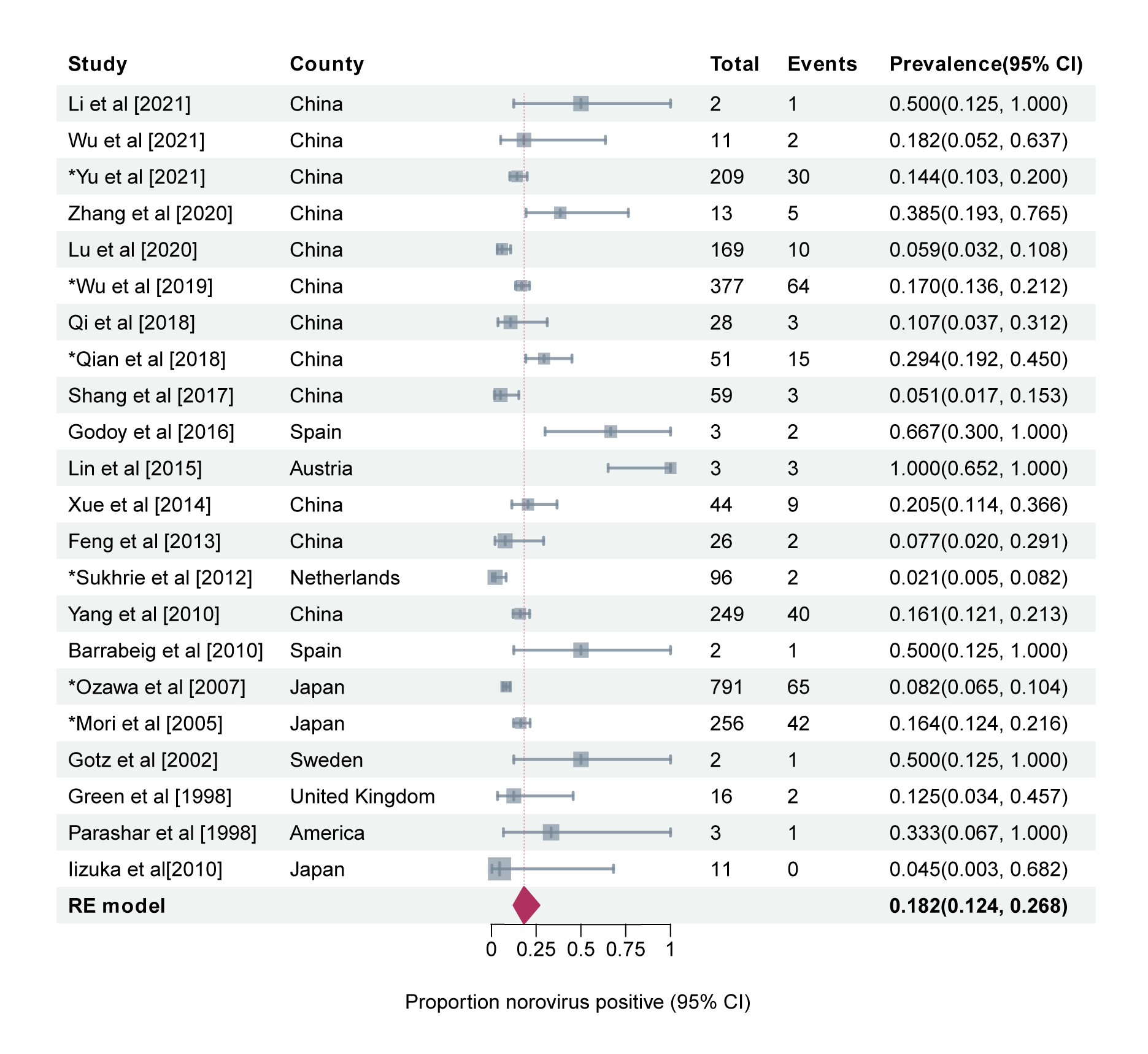
**

**Figure S2.** Funnel plot for assessment of publication bias (Peter's test, *p* = 0.251)

**
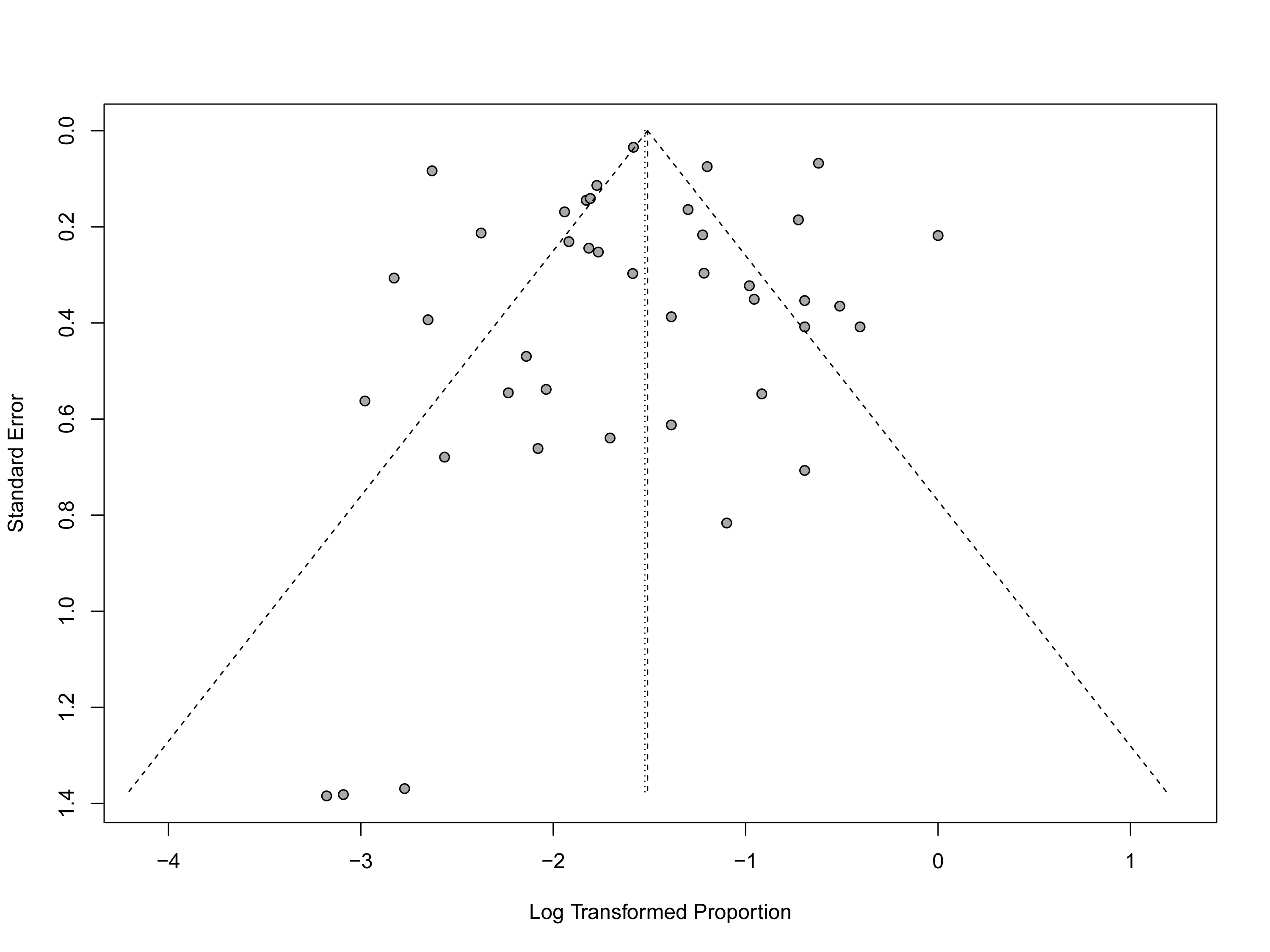
**

**Figure S3.** Sensitivity analysis of meta-analysis (carried out by omitting one study at a time)

**
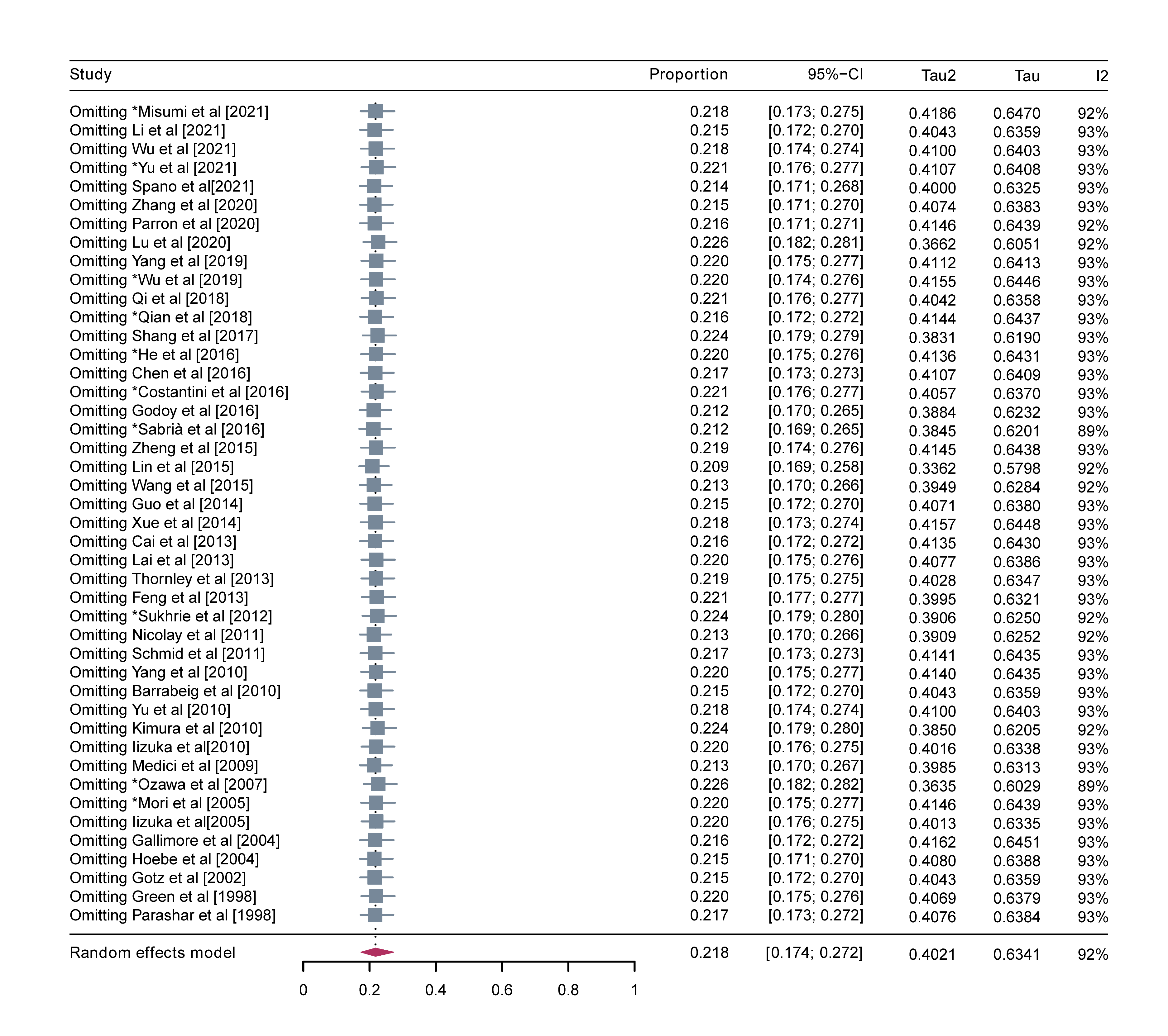
**

**Fig S4.** Subgroup prevalence results of geographic distribution (*I^2^* = 92%, *τ^2^* = 0.4021, *P* < 0.01 test for heterogeneity). Events: Number of NoV-positive asymptomatic individuals. Total: Number of asymptomatic individuals whose samples were detected. *Studies with prevalence were calculated in *N* outbreaks (*N* > 1).

**
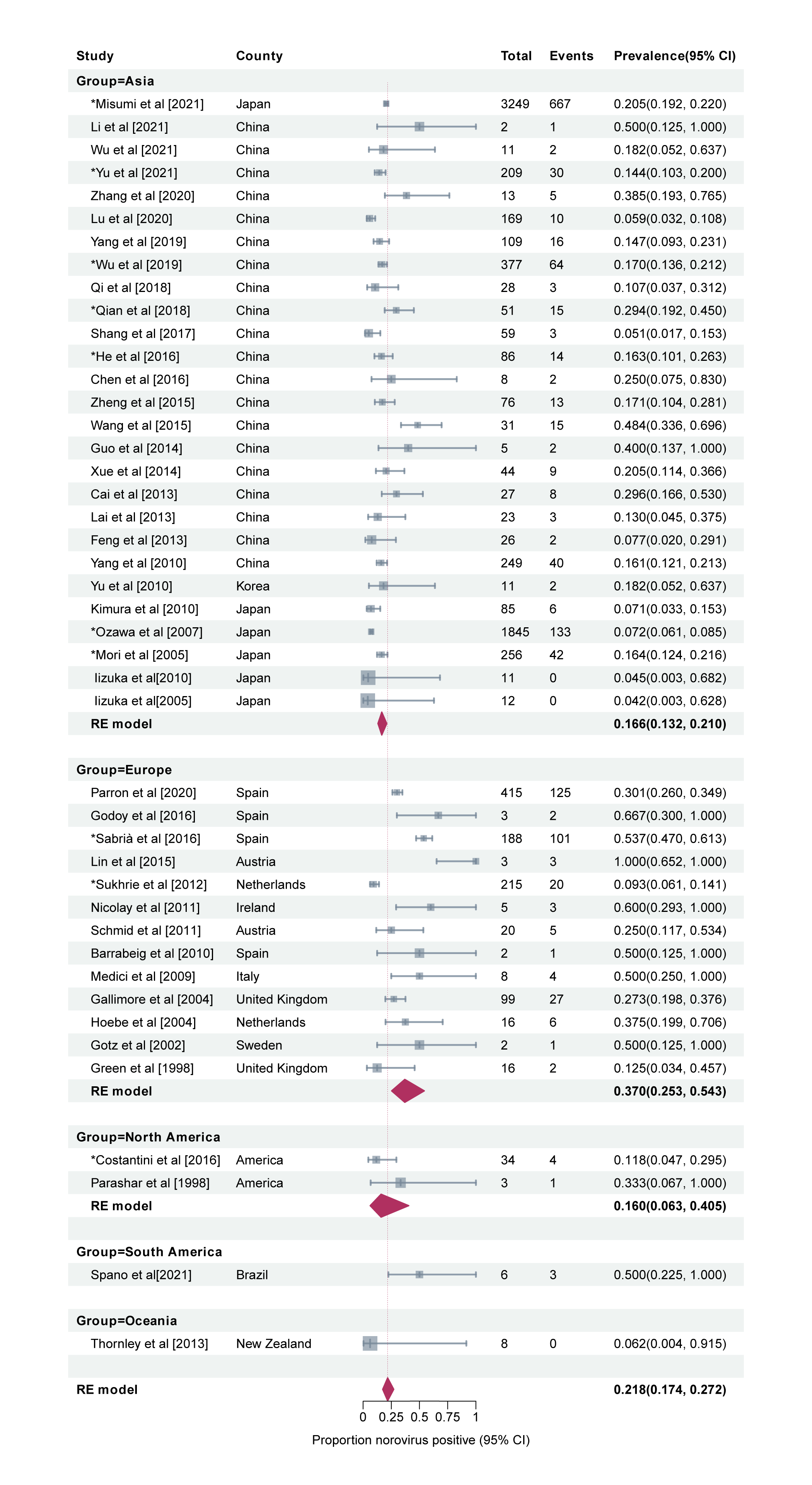
**

**Fig S5.** Subgroup prevalence results of outbreaks settings (*I^2^* = 92%, *τ^2^* = 0.5128, *P* < 0.01 test for heterogeneity). Events: Number of NoV-positive asymptomatic individuals. Total: Number of asymptomatic individuals whose samples were detected. *Studies with prevalence were calculated in *N* outbreaks (*N* > 1).

**
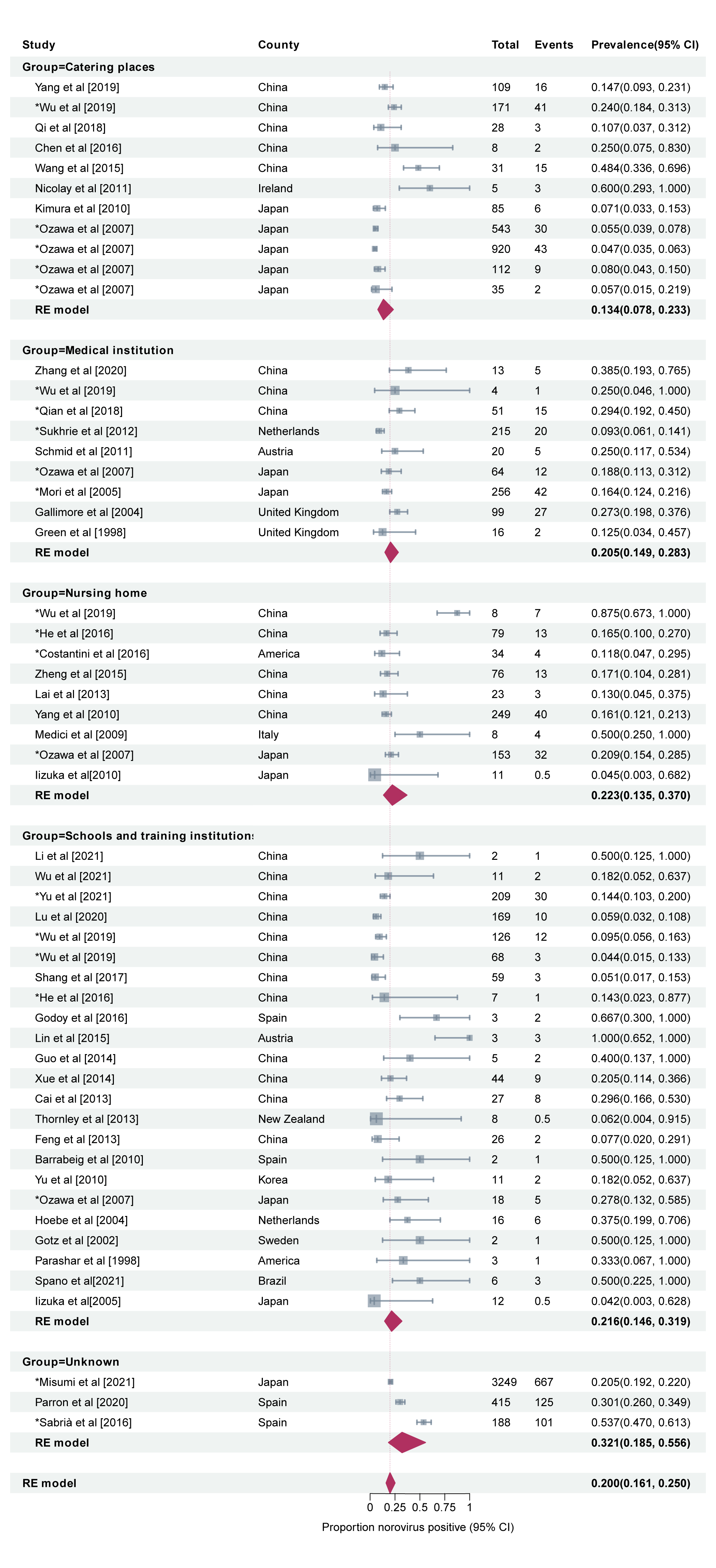
**

**Fig S6.** Subgroup prevalence results of outbreaks seasons (*I^2^* = 90%, *τ^2^* = 0.3550, *P* < 0.01 test for heterogeneity). Events: Number of NoV-positive asymptomatic individuals. Total: Number of asymptomatic individuals whose samples were detected. *Studies with prevalence were calculated in *N* outbreaks (*N* > 1).

**
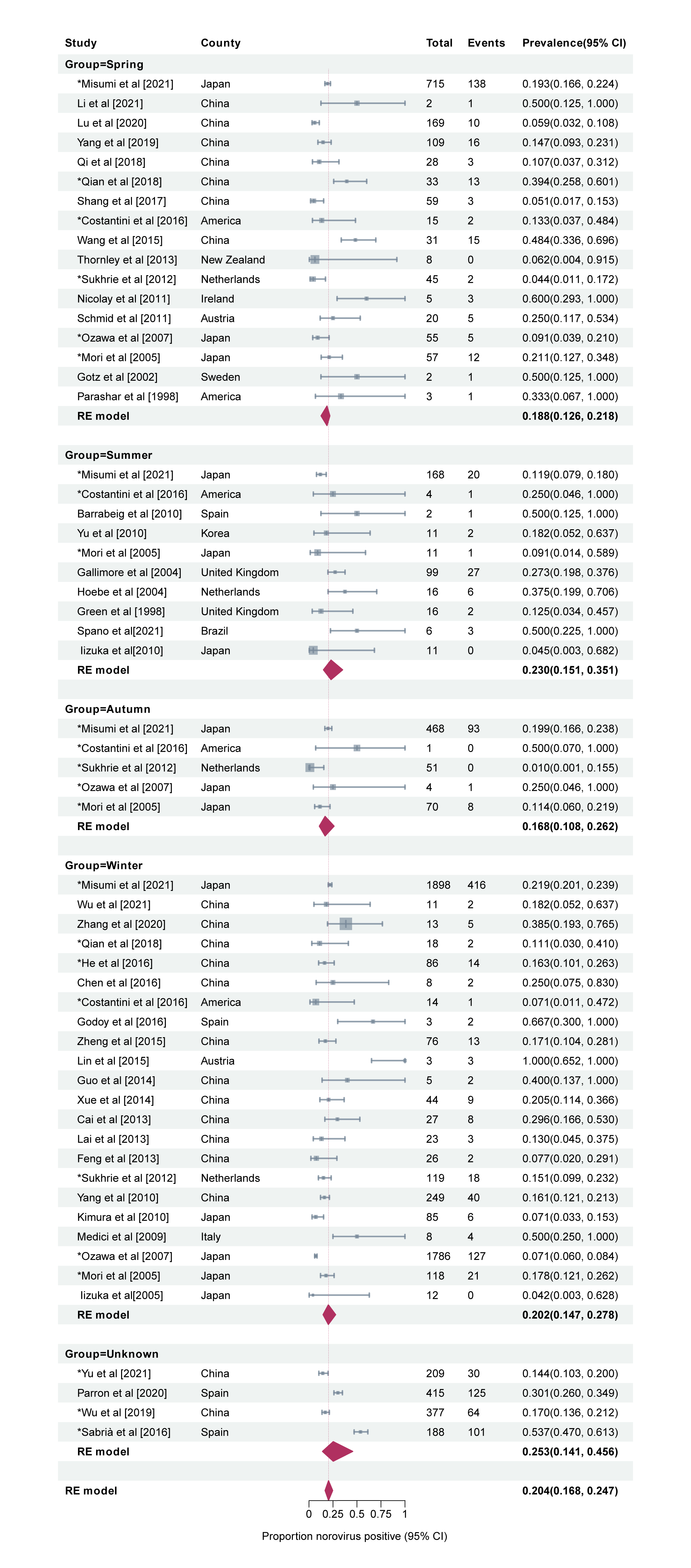
**

**Fig S7.** Subgroup prevalence results of transmission routes (*I^2^* = 92%, *τ^2^* = 0.3992, *P* < 0.01 test for heterogeneity). Events: Number of NoV-positive asymptomatic individuals. Total: Number of asymptomatic individuals whose samples were detected. *Studies with prevalence were calculated in *N* outbreaks (*N* > 1).

**
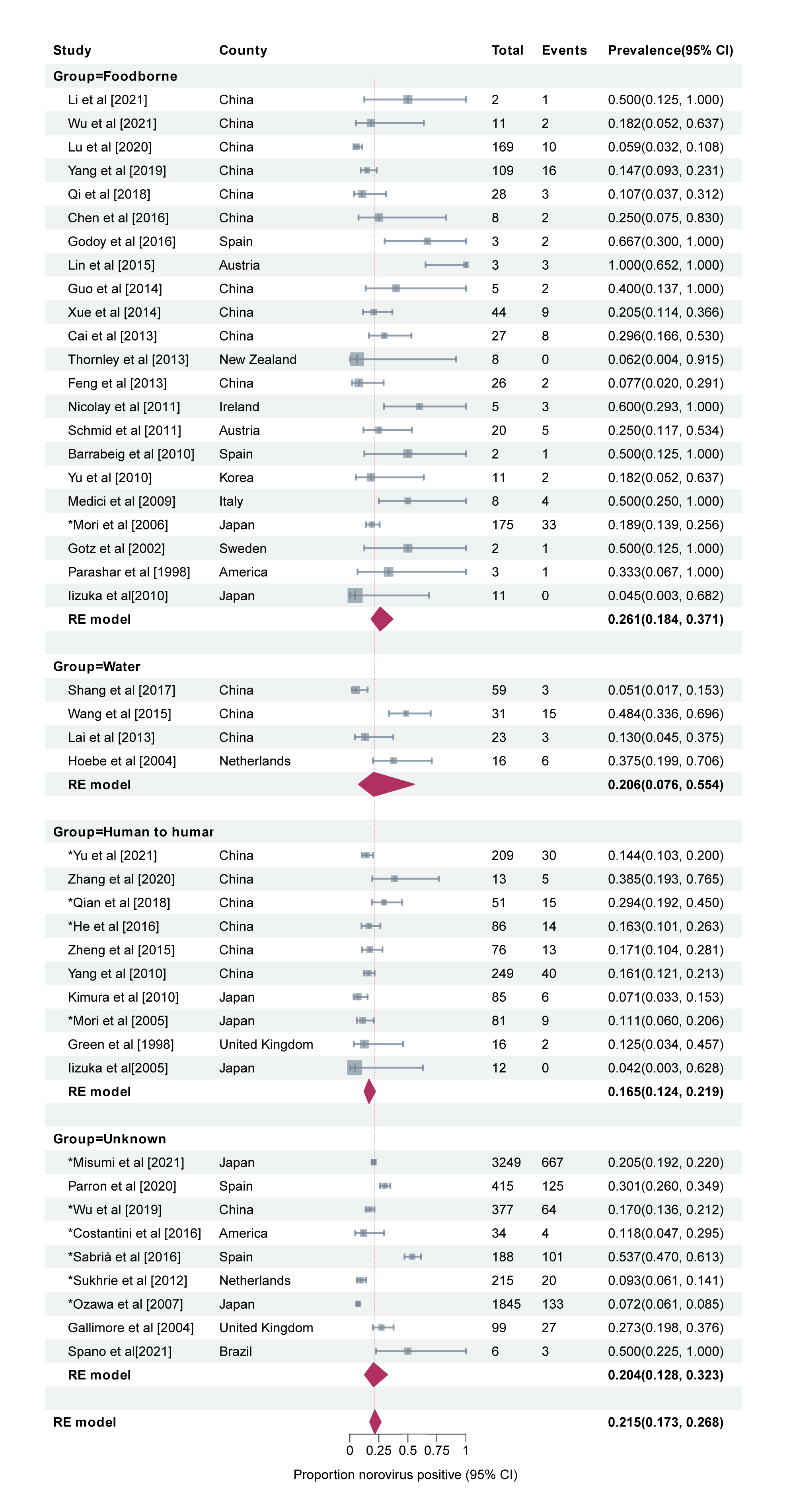
**

**Fig S8.** Subgroup prevalence results of samples types (*I^2^* = 92%, *τ^2^* = 0.4021, *P* < 0.01 test for heterogeneity). Events: Number of NoV-positive asymptomatic individuals. Total: Number of asymptomatic individuals whose samples were detected. *Studies with prevalence were calculated in *N* outbreaks (*N* > 1).

**
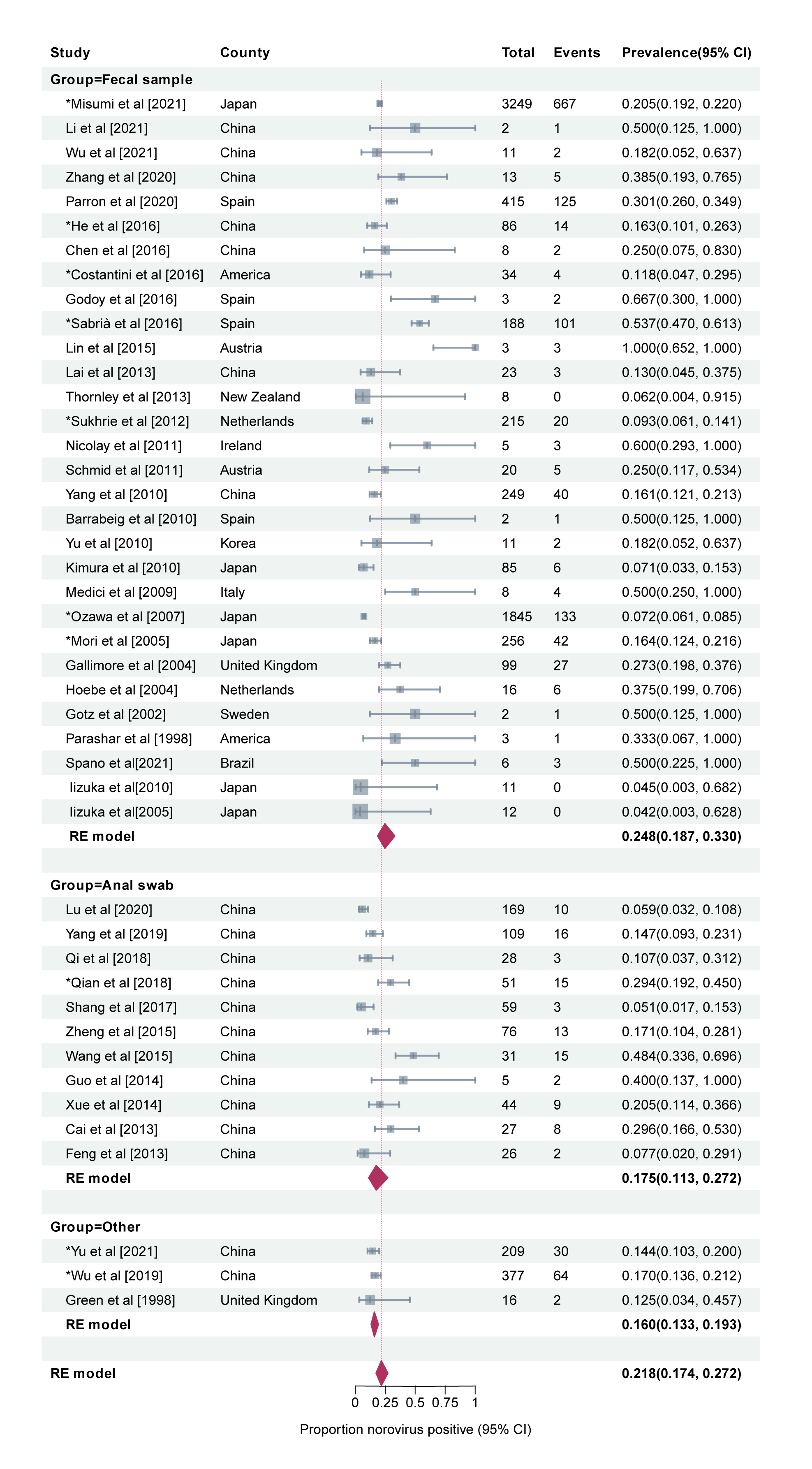
**

**Fig S9.** Subgroup prevalence results of [norovirus](javascript:;) genotypes (*I^2^* = 92%, *τ^2^* = 0.3895, *P* < 0.01 test for heterogeneity). Events: Number of NoV-positive asymptomatic individuals. Total: Number of asymptomatic individuals whose samples were detected. *Studies with prevalence were calculated in *N* outbreaks (*N* > 1).

**
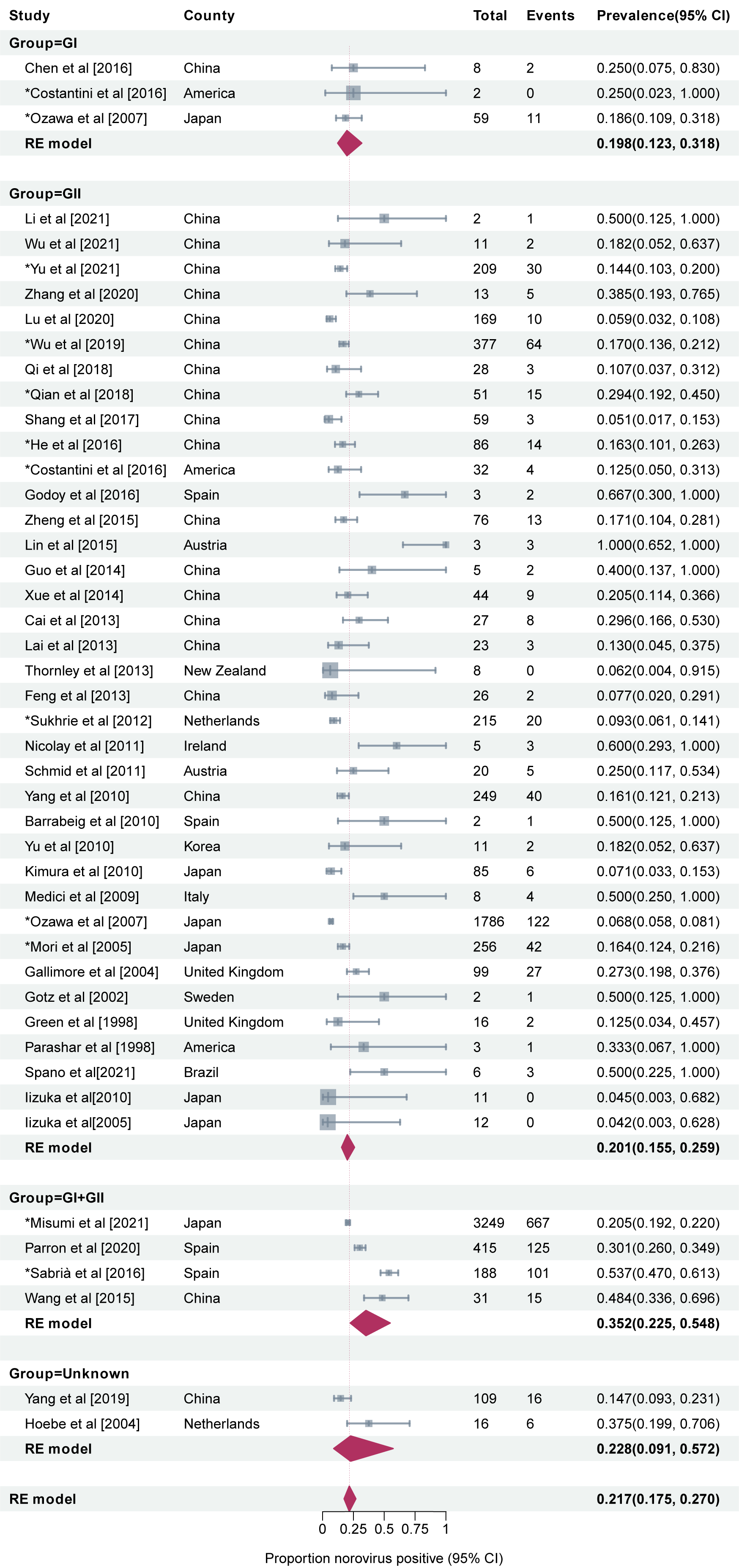
**

**Fig S10.** Subgroup prevalence results of subjects' occupations (*I^2^* = 92%, *τ^2^* = 0.3979, *P* < 0.01 test for heterogeneity). Events: Number of NoV-positive asymptomatic individuals. Total: Number of asymptomatic individuals whose samples were detected. *Studies with prevalence were calculated in *N* outbreaks (*N* > 1).

**
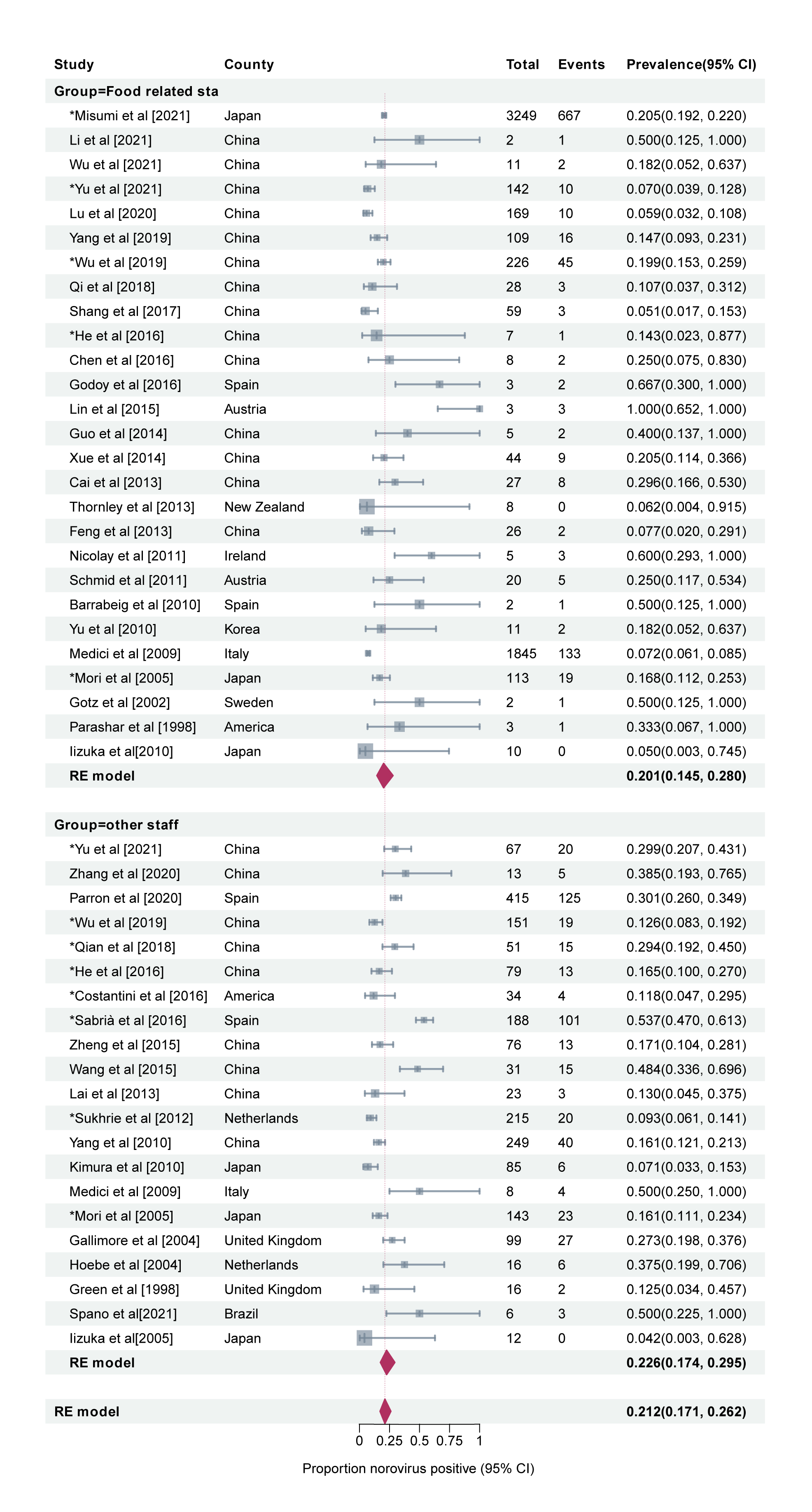
**

**Fig S11.** Subgroup prevalence results of subjects' age (*I^2^* = 92%, *τ^2^* = 0.3936, *P* < 0.01 test for heterogeneity). Events: Number of NoV-positive asymptomatic individuals. Total: Number of asymptomatic individuals whose samples were detected. *Studies with prevalence were calculated in *N* outbreaks (*N* > 1).

**
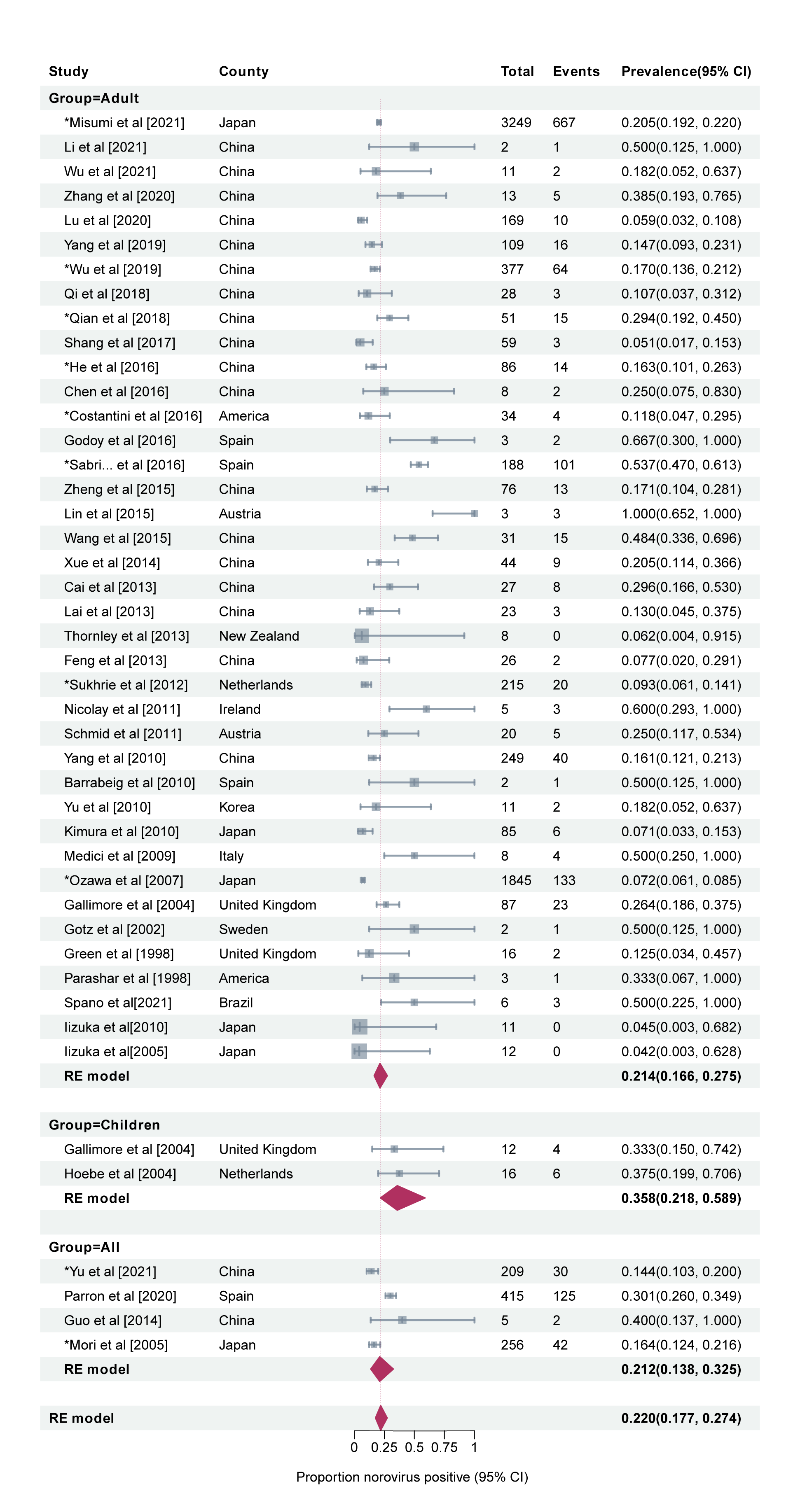
**

**Fig S12.** Subgroup prevalence results of per capita national income (*I^2^* = 92%, *τ^2^* = 0.4021, *P* < 0.01 test for heterogeneity). Events: Number of NoV-positive asymptomatic individuals. Total: Number of asymptomatic individuals whose samples were detected. *Studies with prevalence were calculated in *N* outbreaks (*N* > 1).

**
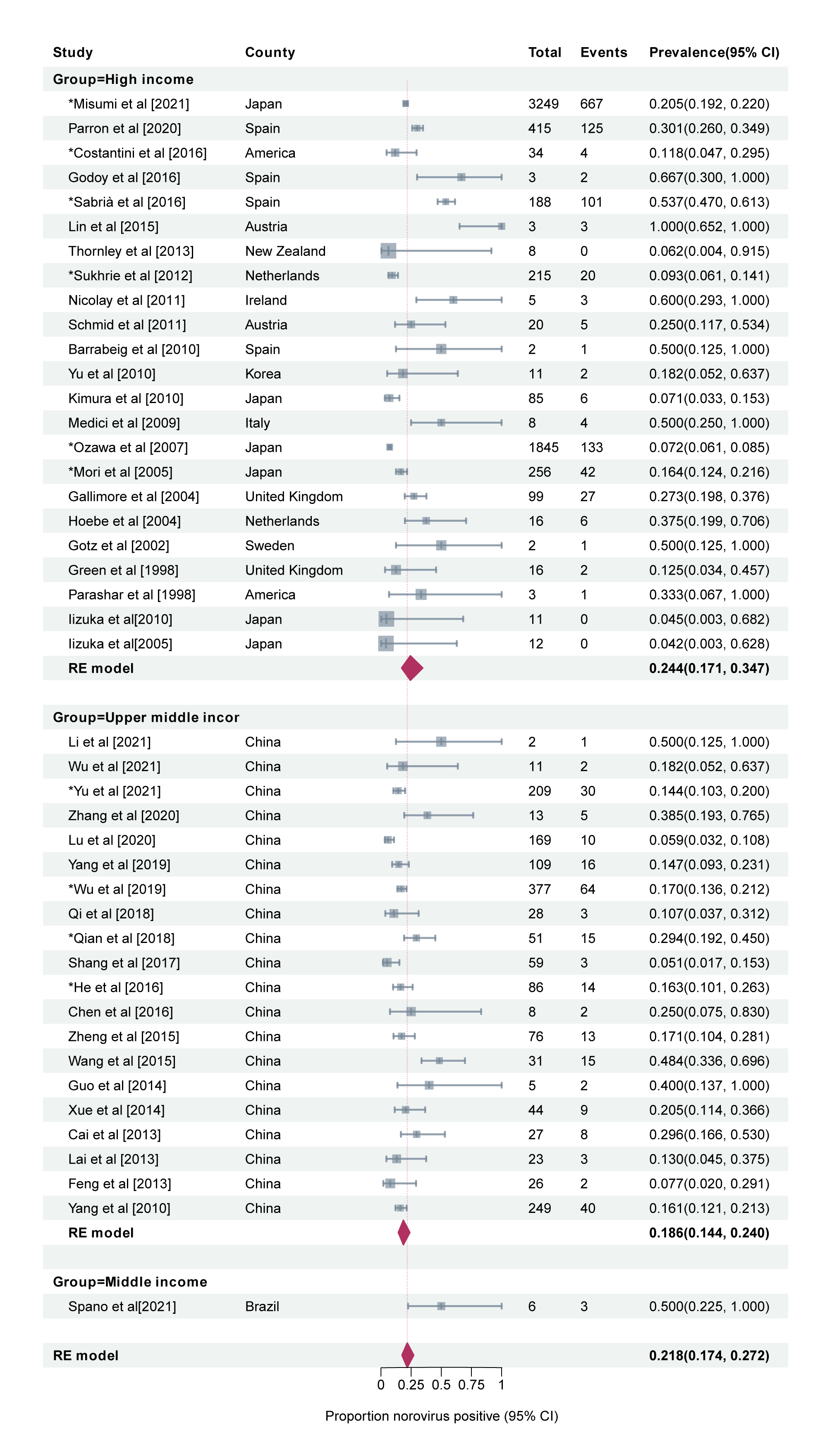
**

**Fig S13.** Subgroup prevalence results of case definition (*I^2^* = 92%, *τ^2^* = 0.4021, *P* < 0.01 test for heterogeneity). Events: Number of NoV-positive asymptomatic individuals. Total: Number of asymptomatic individuals whose samples were detected. *Studies with prevalence were calculated in *N* outbreaks (*N* > 1).

**
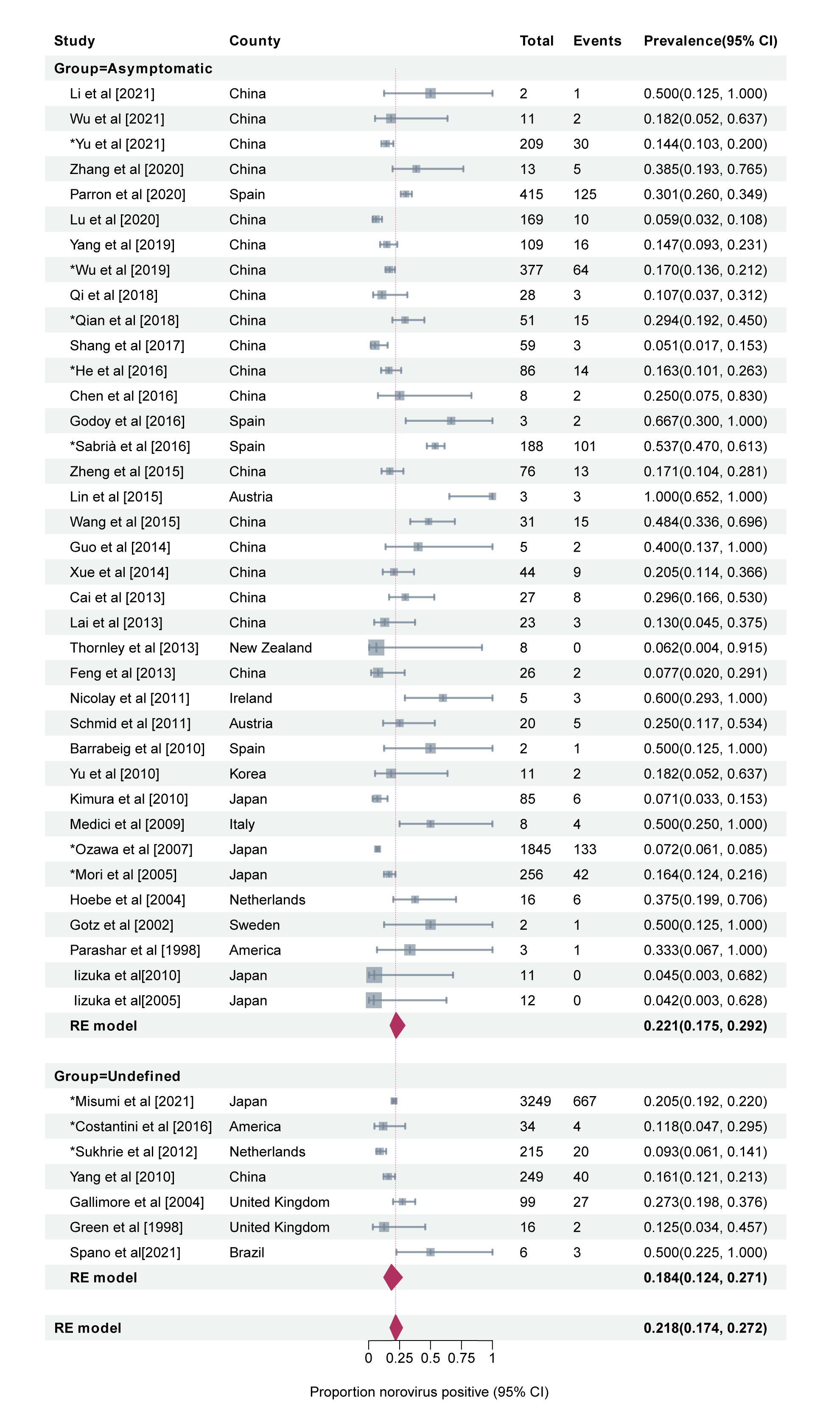
**

**Included Articles**

1. Misumi M, Nishiura H. Long-term dynamics of *Norovirus* transmission in Japan, 2005-2019. PeerJ. 2021;9:e11769. doi: 10.7717/peerj.11769.
2. Spano LC, Guerrieri CG, Volpini LPB, et al. EHEC O111:H8 strain and norovirus GII.4 Sydney P16. causing an outbreak in a daycare center, Brazil, 2019. BMC Microbiol. 2021;21(1):95. doi: 10.1186/s12866-021-02161-x.
3. Li Y, Fan X, Yu G, et al. An acute gastroenteritis outbreak associated with breakfast contaminated with norovirus by asymptotic food handler at a kindergarten in Shenzhen, China. BMC Infect Dis. 2021;21(1):54. doi: 10.1186/s12879-021-05762-z.

4. Wu XW, Wang HW, Ma HL, et al. A foodborne gastroenteritis outbreak caused by Norovirus GII.2. Chinese Journal of Food Hygiene, 2021; 33(2): 234-237. doi: 10.13590/j.cjfh.2021.02.020.

5. Yu XN, Yi LJ, Zhang J, et al. Epidemiological characteristics of norovirus infectious diarrhea in Hongkou District, Shanghai from 2017 to 2019. Chinese Journal of Environmental & Occupational Medicine. 2021; 38(5): 511-516. doi: 10.13213/j.cnki.jeom.2021.20462.

6. Zhang XF, Chen JR, Song CL, et al. Characterization of a hospital-based gastroenteritis outbreak caused by GII.6 norovirus in Jinshan, China. Epidemiol Infect. 2020;148:e289. doi: 10.1017/S0950268820002538.

7. Parrón I, Barrabeig I, Alseda M, et al, The Working Group For The Study Of Acute Gastroenteritis Outbreaks In Catalonia. Involvement of Workers in Closed and Semiclosed Institutions in Outbreaks of Acute Gastroenteritis Due to Norovirus. Viruses. 2020;12(12):1392. doi: 10.3390/v12121392.

8. Lu Y, Ma M, Wang H, et al. An outbreak of norovirus-related acute gastroenteritis associated with delivery food in Guangzhou, southern China. BMC Public Health. 2020;20(1):25. doi: 10.1186/s12889-019-8117-y.

9. Yang J, Zhao XY, Liu SM. Analysis on the duration of intestinal detoxification in restaurant employee infected with Norovirus during the outbreak. Zhonghua Liu Xing Bing Xue Za Zhi. 2019;40(12):1560-1562. doi: 10.3760/cma.j.issn.0254-6450.

10. Wu QS, Xuan ZL, Liu JY, et al. Norovirus shedding among symptomatic and asymptomatic employees in outbreak settings in Shanghai, China. BMC Infect Dis. 2019;19(1):592. doi: 10.1186/s12879-019-4205-y.

11. Qi L, Xiang X, Xiong Y, et al. Outbreak of Acute Gastroenteritis Caused by Norovirus Genogroup II Attributed to Contaminated Cold Dishes on a Cruise Ship in Chongqing, China, 2017. Int J Environ Res Public Health. 2018;15(12):2823. doi: 10.3390/ijerph15122823.

12. Qian ZY, Zhang YW, Yan BQ, et al. Epidemiologic characteristics of 12 cases of acute gastroenteritis outbreaks caused by Norovirus in healthcare facilities in Xuhui District,Shanghai during 2008-2017. Chinese Journal of Nosocomiology. 2018; 28(11): 1633-1636,1653.

13. Shang X, Fu X, Zhang P, et al. An outbreak of norovirus-associated acute gastroenteritis associated with contaminated barrelled water in many schools in Zhejiang, China. PLoS One. 2017;12(2):e0171307. doi: 10.1371/journal.pone.0171307.

14. He Y, Jin M, Chen K, et al. Gastroenteritis Outbreaks Associated with the Emergence of the New GII.4 Sydney Norovirus Variant during the Epidemic of 2012/13 in Shenzhen City, China. PLoS One. 2016;11(11):e0165880. doi: 10.1371/journal.pone.0165880.

15. Chen MY, Chen WC, Chen PC, et al. An outbreak of norovirus gastroenteritis associated with asymptomatic food handlers in Kinmen, Taiwan. BMC Public Health. 2016;16:372. doi: 10.1186/s12889-016-3046-5.

16. Costantini VP, Cooper EM, Hardaker HL, et al. Epidemiologic, Virologic, and Host Genetic Factors of Norovirus Outbreaks in Long-term Care Facilities. Clin Infect Dis. 2016;62(1):1-10. doi: 10.1093/cid/civ747.

17. Godoy P, Alsedà M, Bartolomé R, et al. Norovirus gastroenteritis outbreak transmitted by food and vomit in a high school. Epidemiol Infect. 2016;144(9):1951-8. doi: 10.1017/S0950268815003283.

18. Sabrià A, Pintó RM, Bosch A, et al. Norovirus shedding among food and healthcare workers exposed to the virus in outbreak settings. J Clin Virol. 2016;82:119-125. doi: 10.1016/j.jcv.2016.07.012.

19. Zheng QM, Zeng HT, Dai CW, et al. Epidemiological investigation of a norovirus GII.4 Sydney outbreak in a China elder care facility. Jpn J Infect Dis. 2015;68(1):70-4. doi: 10.7883/yoken.

20. Lin YC, Hipfl E, Lederer I, et al. A norovirus GII.P21 outbreak in a boarding school, Austria 2014. Int J Infect Dis. 2015;37:25-9. doi: 10.1016/j.ijid.2015.05.021.

21. Wang X, Yong W, Shi L, et al. An outbreak of multiple norovirus strains on a cruise ship in China, 2014. J Appl Microbiol. 2016;120(1):226-33. doi: 10.1111/jam.12978.

22. Guo Z, Huang J, Shi G, et al. A food-borne outbreak of gastroenteritis caused by norovirus GII in a university located in Xiamen City, China. Int J Infect Dis. 2014;28:101-6. doi: 10.1016/j.ijid.2014.06.022.

23. Xue C, Fu Y, Zhu W, et al. An outbreak of acute norovirus gastroenteritis in a boarding school in Shanghai: a retrospective cohort study. BMC Public Health. 2014;14:1092. doi: 10.1186/1471-2458-14-1092.

24. Cai WF, Xie HP, Liu YF, et al. An epidemiological investigation on a food-born outbreak of noroviru caused by Sydney 2012 G II.4 strain. Zhonghua Liu Xing Bing Xue Za Zhi. 2013 ;34(8):804-7.

25. Lai CC, Wang YH, Wu CY, et al. A norovirus outbreak in a nursing home: norovirus shedding time associated with age. J Clin Virol. 2013;56(2):96-101. doi: 10.1016/j.jcv.2012.10.011.

26. Thornley CN, Hewitt J, Perumal L, et al. Multiple outbreaks of a novel norovirus GII.4 linked to an infected post-symptomatic food handler. Epidemiol Infect. 2013;141(8):1585-97. doi: 10.1017/S0950268813000095.

27. Ruan F, Tan AJ, Man TF, et al. Gastroenteritis outbreaks caused by Norovirus genotype II.7 in a college in China (Zhuhai, Guangdong) in 2011. Foodborne Pathog Dis. 2013;10(10):856-60. doi: 10.1089/fpd.2013.1519.

28. Sukhrie FH, Teunis P, Vennema H, et al. Nosocomial transmission of norovirus is mainly caused by symptomatic cases. Clin Infect Dis. 2012;54(7):931-7. doi: 10.1093/cid/cir971.

29. Nicolay N, McDermott R, Kelly M, et al. Potential role of asymptomatic kitchen food handlers during a food-borne outbreak of norovirus infection, Dublin, Ireland, March 2009. Euro Surveill. 2011;16(30):19931.

30. Schmid D, Kuo HW, Hell M, et al. Foodborne gastroenteritis outbreak in an Austrian healthcare facility caused by asymptomatic, norovirus-excreting kitchen staff. J Hosp Infect. 2011;77(3):237-41. doi: 10.1016/j.jhin.2010.11.015.

31. Yang LC, Chiang PC, Huang TH, et al. Residents had an increasing risk of norovirus gastroenteritis infection than health care workers during an outbreak in a nursing home. J Am Med Dir Assoc. 2010;11(8):592-7. doi: 10.1016/j.jamda.2009.12.089.

32. Barrabeig I, Rovira A, Buesa J, et al. Foodborne norovirus outbreak: the role of an asymptomatic food handler. BMC Infect Dis. 2010;10:269. doi: 10.1186/1471-2334-10-269.

33. Yu JH, Kim NY, Koh YJ, et al. Epidemiology of foodborne Norovirus outbreak in Incheon, Korea. J Korean Med Sci. 2010;25(8):1128-33. doi: 10.3346/jkms.2010.25.8.1128.

34. Iizuka S, Oka T, Tabara K, et al. Detection of sapoviruses and noroviruses in an outbreak of gastroenteritis linked genetically to shellfish. J Med Virol. 2010;82(7):1247-54. doi: 10.1002/jmv.21791.

35. Kimura H, Nagano K, Kimura N, et al. A norovirus outbreak associated with environmental contamination at a hotel. Epidemiol Infect. 2011;139(2):317-25. doi: 10.1017/S0950268810000981.

36. Medici MC, Morelli A, Arcangeletti MC, et al. An outbreak of norovirus infection in an Italian residential-care facility for the elderly. Clin Microbiol Infect. 2009;15(1):97-100. doi: 10.1111/j.1469-0691.

37. Ozawa K, Oka T, Takeda N, et al. Norovirus infections in symptomatic and asymptomatic food handlers in Japan. J Clin Microbiol. 2007;45(12):3996-4005. doi: 10.1128/JCM.01516-07.

38. Mori K, Hayashi Y, Sasaki Y, et al. Comparison of the number of Norovirus genome copies in patients and healthy persons. Kansenshogaku Zasshi. 2005;79(8):521-6. Japanese. doi: 10.11150/kansenshogakuzasshi1970.79.521.

39. Iizuka S, Tsunomori Y, Tabara K, et al. An outbreak of mixed infection of enterohemorrhagic Escherichia coli O26:H11 and norovirus genogroup II at a kindergarten in Shimane, Japan. Jpn J Infect Dis. 2005;58(5):329-30.

40. Gallimore CI, Cubitt D, du Plessis N, et al. Asymptomatic and symptomatic excretion of noroviruses during a hospital outbreak of gastroenteritis. J Clin Microbiol. 2004;42(5):2271-4. doi: 10.1128/JCM.42.5.2271-2274.2004.

41. Hoebe CJ, Vennema H, de Roda Husman AM, et al. Norovirus outbreak among primary schoolchildren who had played in a recreational water fountain. J Infect Dis. 2004;189(4):699-705. doi: 10.1086/381534.

42. Götz H, de Jong B, Lindbäck J, et al. Epidemiological investigation of a food-borne gastroenteritis outbreak caused by Norwalk-like virus in 30 day-care centres. Scand J Infect Dis. 2002;34(2):115-21. doi: 10.1080/00365540110080133.

43. Green J, Wright PA, Gallimore CI, et al. The role of environmental contamination with small round structured viruses in a hospital outbreak investigated by reverse-transcriptase polymerase chain reaction assay. J Hosp Infect. 1998;39(1):39-45. doi: 10.1016/s0195-6701(98)90241-9.

44. Parashar UD, Dow L, Fankhauser RL, et al. An outbreak of viral gastroenteritis associated with consumption of sandwiches: implications for the control of transmission by food handlers. Epidemiol Infect. 1998;121(3):615-21. doi: 10.1017/s0950268898001150.
